# Exciton Energy Routing via Defect Networks in hBN/2D Perovskite Hybrids

**DOI:** 10.1021/acsnano.6c03445

**Published:** 2026-07-25

**Authors:** Sara Darbari, Paul Bittorf, Leon Multerer, Fatemeh Chahshouri, Parsa Darman, Pavel Ruchka, Harald Giessen, Masoud Taleb, Yaser Abdi, Nahid Talebi

**Affiliations:** † Institute of Experimental and Applied Physics, Kiel University, 24418 Kiel, Germany; ‡ Faculty of Electrical and Computer Engineering, Tarbiat Modares University, Tehran 1411713116, Iran; § Fourth Physics Institute and Research Center SCoPE, 235782 University of Stuttgart, 70569 Stuttgart, Germany; ∥ Kiel Nano, Surface, and Interface Science KiNSIS, Kiel University, 24118 Kiel, Germany; ⊥ Department of Physics, University of Tehran, Tehran 1439955961, Iran

**Keywords:** 2D perovskite, cathodoluminescence, hBN/RPP heterostructure, exciton, defect, energy transfer

## Abstract

Excitons in two-dimensional Ruddlesden–Popper perovskites (RPPs) exhibit large and tunable binding energies, making them promising candidates for optoelectronic applications. In particular, long-range exciton energy transfer in these materials holds potential for light-harvesting technologies and nanoscale interconnects. Here, using cathodoluminescence spectroscopy, we demonstrate that exciton energy can be transferred over ultralong distances—up to 150 μm—in heterostructures composed of hexagonal boron nitride (hBN) and RPPs. This transfer is enabled by efficient exciton coupling to defect centers in hBN and subsequent defect–defect interactions. This mechanism not only facilitates long-range energy transfer but also leads to enhanced luminescence intensity, narrower emission line widths, extended exciton lifetimes, and reduced electron beam-induced degradation. Owing to the high density of emitters within the hBN layers, the investigated van der Waals heterostructure emerges as a robust and stable hybrid platform. Our findings enable room-temperature excitonic devices with enhanced performance, including quantum transducers, light-harvesting systems, and optoelectronic interconnects.

## Graphical abstract

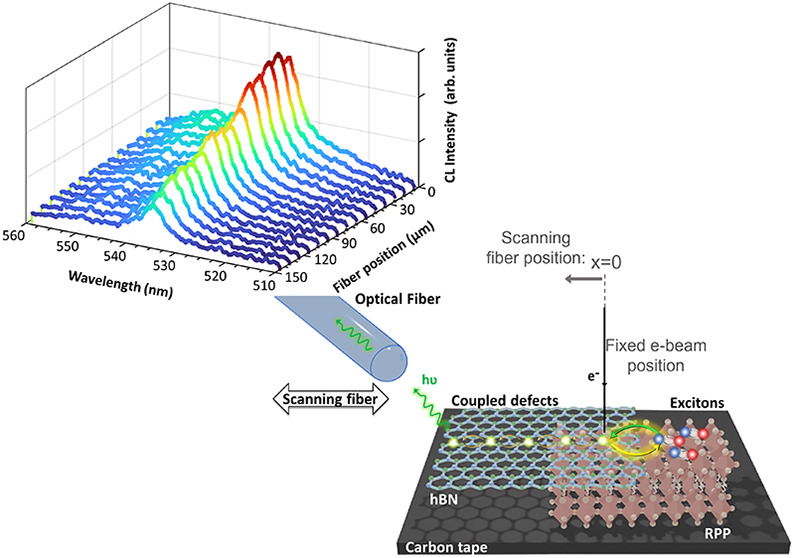

## Introduction

Two-dimensional (2D) perovskites, composed of a stack of 2D quantum well layers and organic spacers, exhibit extraordinary optical properties in addition to higher stability in comparison with their 3D counterparts. These properties have established them as a promising candidate for various optoelectronic and photonic applications.^1–10^ Benefiting from the quantum confinement within the quantum well layers, 2D perovskites exhibit high exciton binding energies at room temperature,^11^ comparable to monolayer 2D transition-metal dichalcogenides (TMDCs).^12–15^ This excitonic behavior of 2D perovskites makes them a valuable platform to achieve strong light-matter interactions and polaritonic responses.^16–20^

Exciton energy transfer mechanisms in nanomaterials have been extensively explored through various mechanisms, including Förster resonant energy transfer,^21–28^ Dexter energy transfer,^29–32^ incoherent photon-mediated energy transfer,^33,34^ and coherent polariton formation.^16,18,35–40^ Here, we demonstrate that there exists another distinguished mechanism, particularly in heterostructures of perovskite and hexagonal boron nitride (hBN); i.e., exciton energy transfer mediated through coupling of excitons to a network of localized defects in hBN.

HBN is one of the most stable and optically transparent encapsulating 2D materials with a large band gap.^41–43^ Moreover, hBN itself hosts multiple classes of phonon-assisted quantum-emitting defects.^44–48^ A variety of these emitters demonstrate photon antibunching, which is a prerequisite for the use of single-photon emitters in quantum optical networks. In addition, radiation damage and degradation of RPPs caused by electron beams in characterization techniques such as cathodoluminescence (CL) spectroscopy can be reduced by hBN-assisted encapsulation.^49–51^

Here, we realize heterostructures of mechanically exfoliated hBN and 2D Ruddlesden–Popper perovskite (RPP), (BA)_2_PbI_4_, where BA is butyl ammonium and the number of PbI_4_-octahedron monolayers is *n* = 1. All structures are positioned on the carbon tape as a substrate attached to a scanning electron microscope stub, even when not directly mentioned. Utilizing CL spectroscopy as a powerful technique for exploring the photon statistics of the emissions from individual defects and clusters in several kinds of 2D materials as well as exploring the exciton physics of semiconductors,^52–58^ we study the optical response of the realized hBN/RPP heterostructures on carbon tape (C). Our results show an enhanced CL intensity, reduction in the line width broadening of the emission peak, increased decay time for the hBN/RPP heterostructures in comparison with RPPs, which is attributed to the efficient exciton–defect coupling at the interface of hBN and RPP. Significantly retarded degradation of RPPs is also achieved in hBN/RPP heterostructures during exposure to an electron beam, allowing reliable CL measurements at a low-current regime. Moreover, benefiting from a newly developed 3D-printed fiber-based CL setup,^59^ we study the exciton energy transfer mechanisms in hBN/RPP heterostructures via changing the collecting fiber distance from the electron beam excitation location. This study reveals an ultralong-range incoherent energy transfer, in contrast with exciton polaritons,^12,17^ through extruded parts of hBN in the hBN/RPP heterostructure over distances of about 150 μm, which is attributed to defect–defect coupling and photon recycling through the defect emitters in hBN. Our findings establish hBN/RPP heterostructures as hybrid van der Waals systems that exhibit enhanced luminescence and ultralong-range exciton energy transfer. They are also promising candidates for room-temperature excitonic applications.

## Results and Discussion

### Cathodoluminescence Enhancement and Decay Rate of the Excitons in hBN/RPP Heterostructures

To explore the optical properties of the hBN/RPP heterostructure, we first utilize a fiber-collected CL spectroscopy technique developed in our group,^59^ in which the heterostructure is excited by the electron beam and the emitted radiation is collected by an integrated multimode optical fiber with a 3D-printed lens at its facet. The collected CL light is guided into the analyzing path, as depicted in Figure 1a. In this configuration, the sample and the fiber are mounted onto individual three-axis piezo stages for precise in situ positioning inside a scanning electron microscope (SEM) chamber. In the first measurement, the fiber focal point has been adjusted and fixed on the electron beam impact position on the sample, while the sample stage is moved in the *x*–*y* plane to expose different sample parts to the electron beam (Figure 1a).

**Figure 1:**
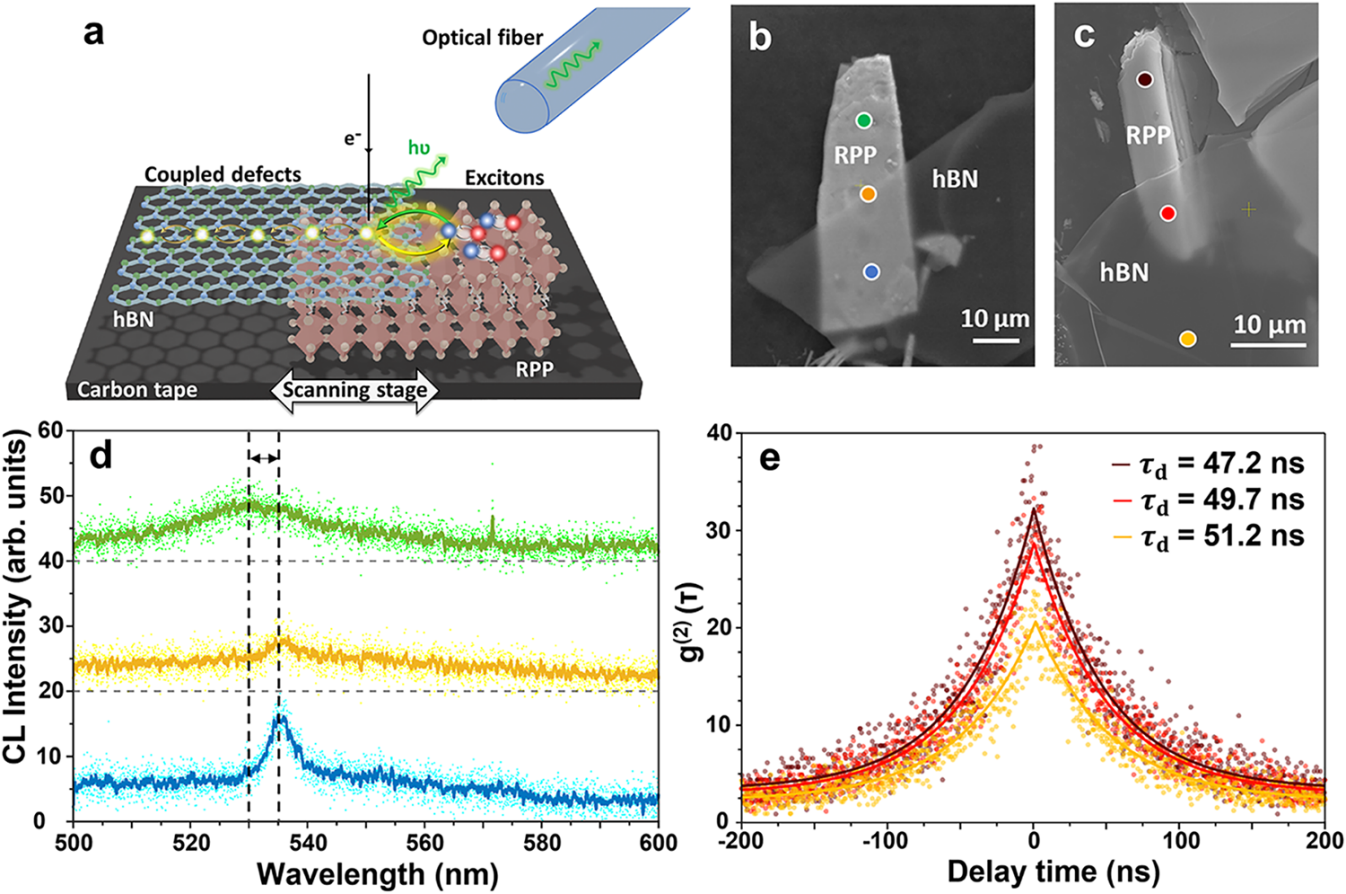
Enhanced CL peak and lifetime measurements facilitated by a fiber-assisted CL spectroscopy system. (a) Schematic of the fiber-collected CL spectroscopy setup used for the hBN/RPP heterostructure. The emitted light is collected via a fiber with a 3D-printed lens on its facet and guided into the analyzing path. The focal point of the lens is fixed at the electron beam incident point on the sample, and the sample lateral position can be changed by a scanning stage under the electron beam. (b, c) SEM secondary electron images of the investigated hBN/RPP heterostructures, wherein colored circles show the electron impact positions. (d) Fiber-collected CL spectra for electron positions marked by the correspondingly colored circles in (b). (e) Second-order autocorrelation measurements (g^(2)^(τ)) from different electron impact positions, marked by the correspondingly colored circles in (c), revealing electron beam-induced synchronization of emitters and photon bunching. Associated lifetimes of excitations at different areas of the sample are depicted as well.

The CL spectra of different locations on an hBN/RPP heterostructure have been acquired by focusing the electron beam excitation and the collecting fiber on the RPP flake (green circle), the hBN edge on the RPP flake (orange circle), and the hBN/RPP heterostructure (blue circle) (Figure 1b). The acceleration voltage was 20 kV, and the current was fixed at 140 pA in the low-current regime, measured at the sample position using an implemented Faraday cup.

The measured CL spectrum exhibits a broad excitonic peak with the central wavelength of 530 nm and a full wave half maximum (FWHM) of 25 nm for the pure RPP flake (green spectrum in Figure 1d corresponding to the green circle in Figure 1b), whereas a 5 nm red shift and 75% reduction in the bandwidth are observed for the electron beam positioned at the hBN edge (orange spectrum in Figure 1d corresponding to the electron position marked with orange circle in Figure 1b). The CL peak is further enhanced and becomes narrower when the electron beam traverses the heterostructure farther away from the edge (blue spectrum in Figure 1d corresponding to the electron position marked by the blue circle in Figure 1b). In all cases where the emission from defects is pronounced, phonon sidebands are observed as well, confirming the prominent role of defects in mediating the exciton and defect interactions, as will be elaborated further below (see Supporting Information Note 8 for more information). The presented CL measurements and the corresponding observed enhancements in the excitonic peak of the hBN/RPP heterostructure are in analogy with the previously reported enhancements in hBN/TMDCs,^60–68^ where the observed phenomenon has been attributed to the transfer of the excited electrons and holes in hBN into the 2D TMDC as the narrow band gap material in the heterostructure.^60,61^ Furthermore, in other studies, the reduced line width broadening in hBN-encapsulated TMDCs has been attributed to the reduced surface roughness, reduced substrate-induced charge trapping, and protection from electron beam irradiation in TMDCs because of encapsulating hBN flakes.^65,67^ However, such mechanisms cannot explain the observations reported here, that is, the long-range exciton energy transfer in the extruded parts of the hBN flake in the hBN/RPP heterostructure, as will be shown below. Here, we attribute the observed red shift and reduced bandwidth dominantly to the phonon-assisted decay of the excited excitons in RPP to the localized and narrow bandwidth defect states in hBN, where the resonant energy of the latter is near to the RPP exciton wavelength. The proposed mechanism is elaborated in Figure 4c.

The lifetime of the excited carriers in hBN/RPP heterostructure is measured using a fiber-based Hanbury Brown and Twiss (HBT) intensity interferometer, allowing us to acquire the second-order autocorrelation function *g*_CL_^(2)^(τ) of the measured CL intensity signal *I*_CL_(*t*) (Figure 1c).^63,69–71^ This helps us to gain insights into the coherent and incoherent interaction processes of high-energy electrons within the heterostructures, where multiple quantum states are excited by each incoming electron. Since the excitation happens on extremely short interaction times of less than 1 fs, an inherent time synchronization is established between the emitters, resulting in an observed bunching effect. These excitations diffuse and decay radiatively over time;^72^ hence, the measured second-order autocorrelation function (*g*_CL_^(2)^(τ)) displays a bunching effect at zero delay (*g*_CL_^(2)^(0) ≫ 1). Moreover, the decay rate of the bunching peak reveals the radiative lifetime of the ensembles of emitters. *g*_CL_^(2)^(τ) of the hBN/RPP heterostructure at different electron beam excitation positions is measured (shown by colored circles in Figure 1c), the results of which are plotted in Figure 1e by the corresponding colors. For each sample position, the model function was fitted to the experimental data.^71^

1 $$g_{\mathrm{CL}}^{\left(2\right)}=1+g_{0}\cdot \exp\left(-\left|\tau \right|/\tau_{d}\right)$$

where *g*_0_ represents the amplitude of *g*_CL_^(2)^ at the delay time of τ = 0 and τ_d_ is the decay time of the excited state, or its lifetime. At all electron beam excitation locations, the bunching peaks exhibit similar magnitudes of 32.5, 28, and 20.5 for the RPP (brown curve), the hBN/RPP heterostructure (red curve), and the extruded part of hBN (orange curve), respectively. These values are significantly higher than the reported bunching peaks for hBN/TMDC heterostructures at a comparable current level.^51^ However, the peak of *g*_CL_^(2)^ is the smallest when the electron beam excites the extruded part of hBN (orange circle in Figure 1c), away from the underlying edge of RPP in the hBN/RPP heterostructure. Moreover, the measured decay time for the extruded part of the hBN flake (51.2 ns) is slightly longer than that for the hBN/RPP (49.7 ns), and the latter is longer than that for the pure RPP (47.2 ns). The observed increased lifetime in Figure 1e is consistent with the previously observed decreased CL peak bandwidth for the hBN/RPP heterostructure in comparison with the RPP in Figure 1d. It is notable that the measured longer decay time, implying the slower annihilation of the excitons in the hBN-passivated RPP, can be attributed to the reduced surface roughness, surface passivation, and protection against electron beam irradiation by the top hBN flake.^65,67^ Nevertheless, the emission lifetimes extracted from our HBT-based measurements show only minor changes between bare RPP and hBN/RPP heterostructures.

This observation is consistent with a transport mechanism in which excitation redistribution through the hBN defect network occurs on time scales shorter than the intrinsic radiative lifetime. In such a regime, energy can propagate over long distances without requiring a pronounced modification of the local spontaneous emission rate.

### Long-Range Exciton Energy Transfer in hBN/RPP Heterostructures via Exciton–Defect–Defect Interactions

Next, we investigate the hBN/RPP heterostructure by a mirror-collected CL spectroscopy system (Figure 2a) at a low current level of 200 pA and an acceleration voltage of 20 kV. In this measurement, a parabolic aluminum-coated mirror efficiently collects and collimates the emitted CL radiation and projects it into the analyzing path.

**Figure 2:**
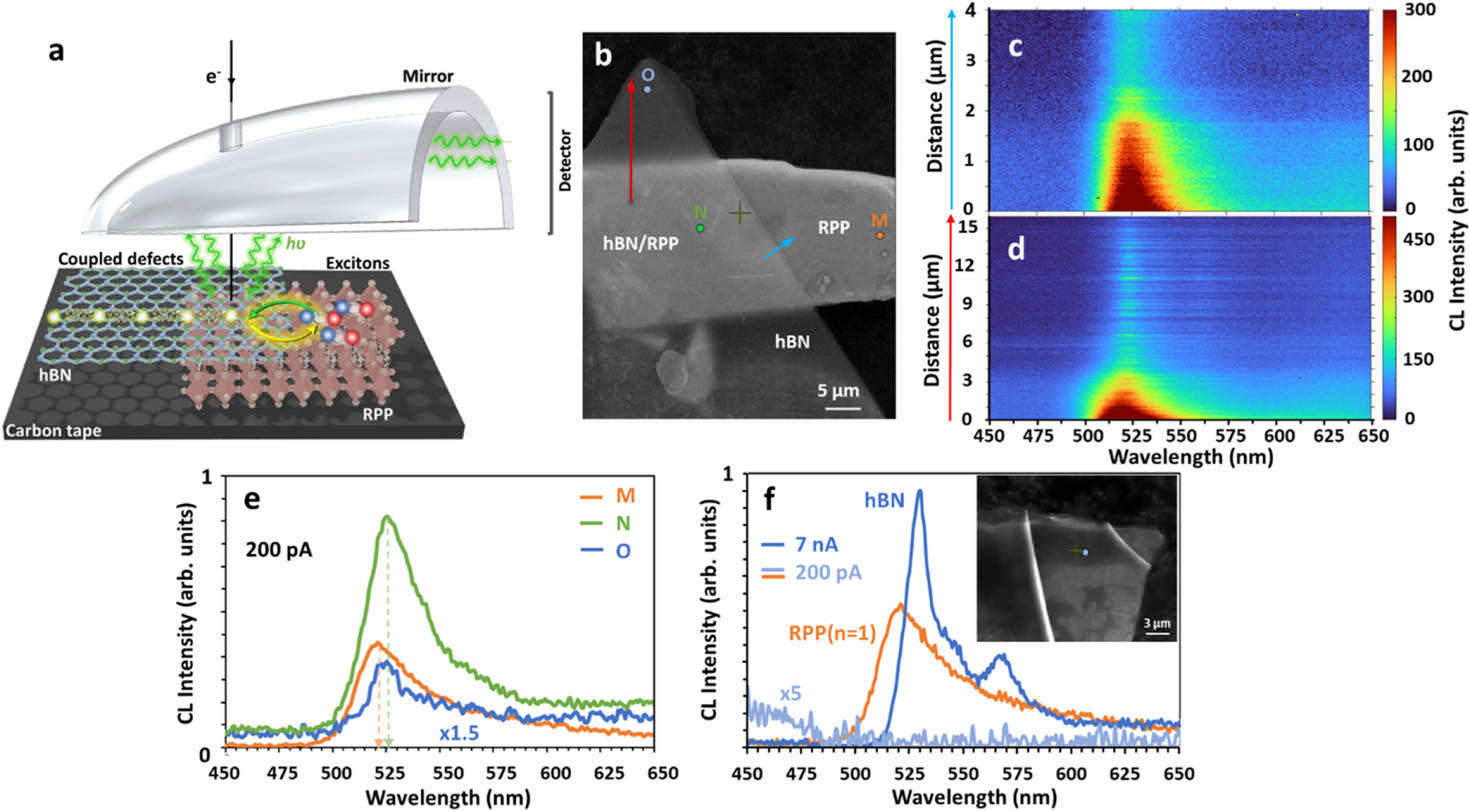
Spatially resolved cathodoluminescence response of the hBN/RPP heterostructure. (a) Schematic demonstration of the mirror-collected CL spectroscopy setup used for the hBN/RPP heterostructure at a current of 200 pA and acceleration voltage of 20 kV. The emitted light is collected by a parabolic mirror and directed toward a spectrometer. (b) SEM image of the investigated hBN/RPP heterostructure, wherein the blue and red arrows show the electron beam scanning paths across the top hBN edge and the underlying RPP edge, respectively. (c, d) Measured CL spectra along the blue (top) and red (bottom) scanning arrows in part (b). (e) Individual CL spectra corresponding to three different spots, marked as ‘M’, ‘N’, and ‘O’ in (b). (f) CL spectra of a pure hBN flake (without RPP) integrated over a large area at the low-current (200 pA) and high-current (7 nA) regimes, superimposed on the CL spectrum of a pure RPP flake at low-current (200 pA) regime. The inset displays the SEM image of the investigated pure hBN flake.

The sample is scanned by the electron beam (blue and red arrows in Figure 2b), while the collecting optics remain at a fixed position. This way, higher spatial resolution is achieved, as compared to our previous results based on the fiber-collected setup, where the stage was moved instead of the electron beam. The measured energy–distance CL intensity maps for scanning the electron beam along the blue and red arrows are displayed in Figure 2c,2d. Figure 2c confirms the enhanced CL excitonic peak at the hBN/RPP heterostructure in comparison with the RPP, revealed by a step-like decrement in CL intensity as electron beam passes the hBN edge over the RPP flake at the distance of about 2 μm. Moreover, while electron beam passes over the edge of the underlying RPP flake at the distance of about 3 μm in Figure 2d, the excitonic peak is significantly decreased but keeps nearly a constant intensity on the extruded part of the top hBN flake up to distances of 15 μm. This observation is attributed to the excited excitons in RPP (*n* = 1) that are coupled to the nearly matched electronic states of defects in hBN. Interestingly, a prominent CL signal is still observed when the electron beam impinges on the extruded part of the top hBN flake far away from the underlying RPP flake. In other words, Figure 2d shows a long-range exciton energy transfer through hBN in the hBN/RPP heterostructure over a distance of several micrometers. Moreover, Figure 2d reveals a spatially discretized profile for the CL emission peak in the extruded hBN after passing RPP edge along the red arrow. This phenomenon, together with the long-range exciton energy transfer originated from the coupling of defects in hBN to the excitons in RPP at the hBN/RPP interface, as will be elucidated in more detail below.

The acquired individual CL spectra in Figure 2e reveal an excitonic peak at 520 nm for the RPP (position ‘M’ in Figure 2b), an enhanced CL peak at 525 nm for the hBN/RPP (*n* = 1) heterostructure (position ‘N’ in Figure 2b),^17,73^ and a weak peak at 525 nm for the extruded part of hBN (position ‘O’ in Figure 2b). Accordingly, the observed CL peaks and the slight red shift of about 5 nm (shown by red and orange dashed arrows in Figure 2e) in the mirror-collected CL peaks of the hBN/RPP heterostructure in comparison with RPP are in accordance with the results of the described fiber-collected CL apparatus in Figure 1d.

It should be mentioned that there exists a fixed wavelength offset of about 10 nm in the utilized fiber-collected setup with respect to the mirror-collected CL spectroscopy apparatus, revealing the agreement between the excitonic CL peaks of RPP and those of the hBN/RPP heterostructure. Additionally, a weak shoulder at around 570 nm is observed beside the main CL peak of the hBN/RPP (*n* = 1) heterostructure, which is attributed to the excitation of phonon sidebands of defects in hBN.^44,47,74^

To elaborate on the proposed exciton–defect coupling in the hBN/RPP heterostructure, the CL spectrum of a pristine hBN flake is illustrated in addition to that of the pristine RPP flake (Figure 2f). The CL spectrum of a pure hBN flake reveals no detectable CL peak at the low-current regime (200 pA), similar to the current used for the RPP and hBN/RPP samples. However, benefiting from the inherent stability of hBN, we increased the beam current to a high-current regime of 7 nA at the same acceleration voltage of 20 kV and acquired a clear CL trace of luminescent defects in the pure hBN with a strong and sharp peak at 530 nm and a weaker second peak at 570 nm, where the latter corresponds to the phonon sidebands. The defects observed here are point defects, as better confirmed in the hyperspectral images shown in Supporting Information Figure 18b,c. These point defects that emit at a stable spectral range with a fixed peak wavelength of 530 nm have a nitrogen vacancy nature, as demonstrated elsewhere with ab initio calculations.^75^

Figure 2f reveals a significant spectral overlap between the excitonic peak at 520 nm in RPP (*n* = 1) and the luminescent defect peak at 530 nm in hBN, in addition to a weaker spectral overlap between the excitons and the second peak of hBN at 570 nm. This study solidifies the proposed efficient exciton–defect coupling as the main reason behind the observed enhanced CL intensity, the slight red shift, and the decreased bandwidth in the hBN/RPP heterostructure. Moreover, this study implies that the hBN defect energy states in this wavelength range are not excited efficiently by an electron beam at a low-current regime of 200 pA. However, coupling to excitons in the hBN/RPP heterostructure enables a strong CL trace at 530 nm even at low-current excitations, highlighting the high efficiency of defect excitations via excitons.

Linear plots of the CL intensity variations along the white arrow over the hBN/RPP heterostructure (inset), at peak wavelengths of 525 and 570 nm, better indicate the long-range energy transfer mechanisms (Figure 3a). As observed, the enhanced excitonic peak intensity at 525 nm on the hBN/RPP heterostructure drops to a lower value by passing across the underlying RPP edge but remains nearly constant over the rest of the scanning length of about 10 μm over the extruded part of hBN. A similar behavior is observed for the CL intensity at 570 nm; however, it has been significantly lower on the heterostructure and drops to comparatively lower intensities than that of the excitonic wavelength by passing across the underlying RPP edge. Furthermore, the intensity fluctuations demonstrated by the green curve in Figure 3a confirm the closely located luminescent centers and the efficient coupling of excitons to defects in the hBN/RPP heterostructure.

**Figure 3:**
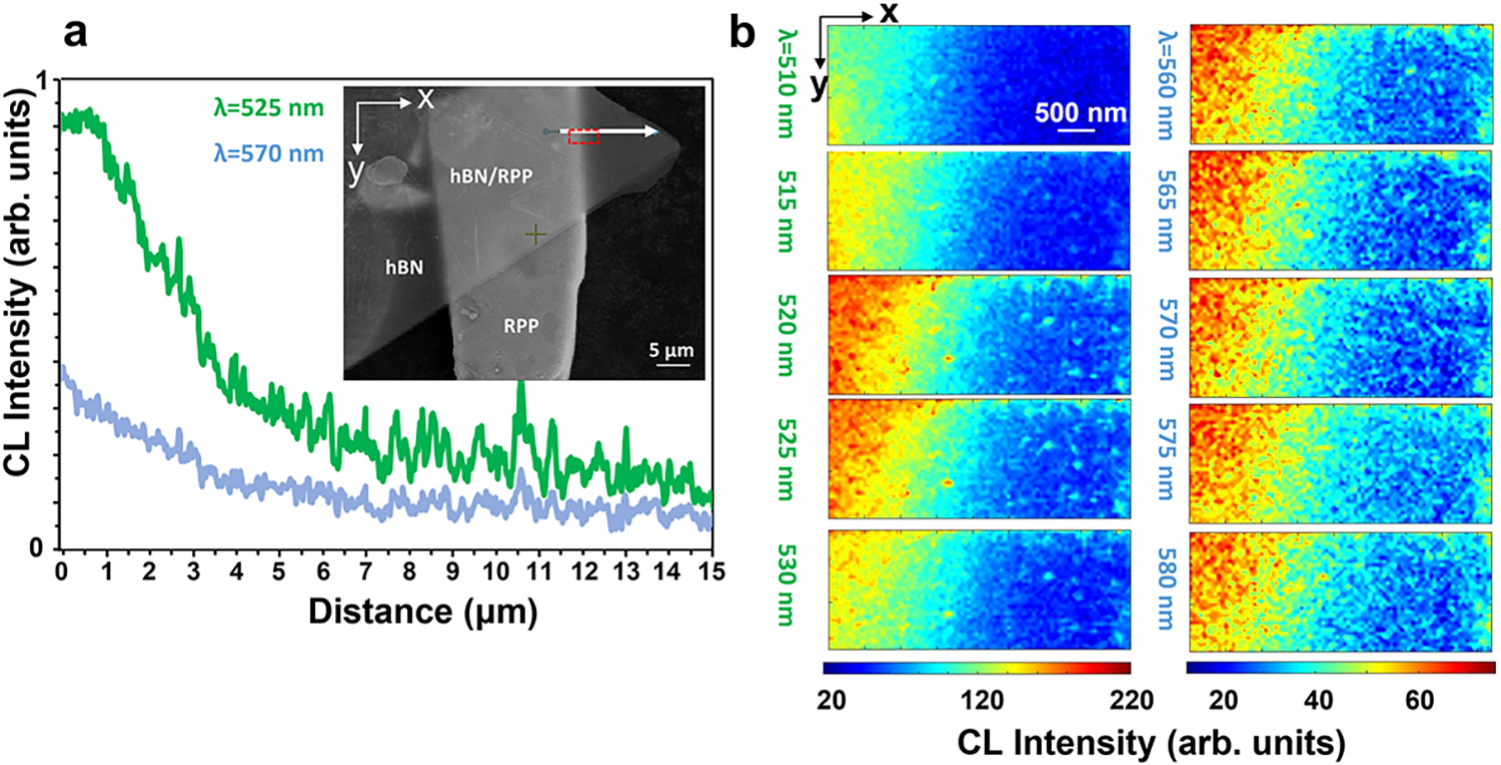
Defects in hBN coupled to excitons. (a) Intensity variations of the CL peaks in Figure 2e, at 525 nm (green curve) and 570 nm (blue curve) versus the electron beam scanning position along the white arrow in the inset. The inset shows the secondary electron SEM image of the investigated hBN/RPP heterostructure. (b) 2D spatial profile of CL peak intensities at wavelengths around 525 and 570 nm, acquired over the dashed red rectangular box in the inset of panel (a).

CL hyperspectral images at the wavelengths of 525 and 570 nm (Figure 3b), captured from the spatial region shown by the red, dashed rectangle in the inset of Figure 3a, better highlight the relevance of the exciton–defect coupling scenario. The first and second columns in Figure 3b display the CL intensity distributions spectrally filtered at selected wavelengths from 510 to 530 nm near the electronic level of defects and at wavelengths of 560 to 580 nm attributed to the phonon sideband, respectively. At 515 nm, local bright spots emerge across the whole scanning area, both on the hBN/RPP (left half of the frame) and on the extruded hBN (right half of the frame), which can be attributed to the efficient excitation of local defects in hBN coupled to the excitons. The observed luminescence and the emerged local bright spots show a highest intensity at around the excitonic wavelength of 520 nm, exhibiting lower intensities at shorter and longer wavelengths. The second column in Figure 3b indicates lower CL intensities with less localized bright spots around 570 nm, following nearly the same spatial distribution around the excitonic wavelength, strengthening the hypothesis of coupling between different luminescent centers associated with defects in hBN and the excitons in RPP.

Furthermore, we explore the long-range exciton energy transfer inside the hBN/RPP heterostructure by the fiber-collected CL spectroscopy technique, benefiting from a local detection scheme, due to the ability to individually move the CL-collecting fiber with a piezo stage, while the electron beam impacts the sample at a fixed position. For this experiment, we investigate an hBN/RPP heterostructure with a large hBN flake positioned on top of an RPP flake, where the size of the latter is approximately 15 × 30 μm^2^ (Figure 4a). The beam current in this measurement is fixed at a low current regime (below 200 pA) and the acceleration voltage is fixed at 20 kV, similar to previous measurements.

**Figure 4:**
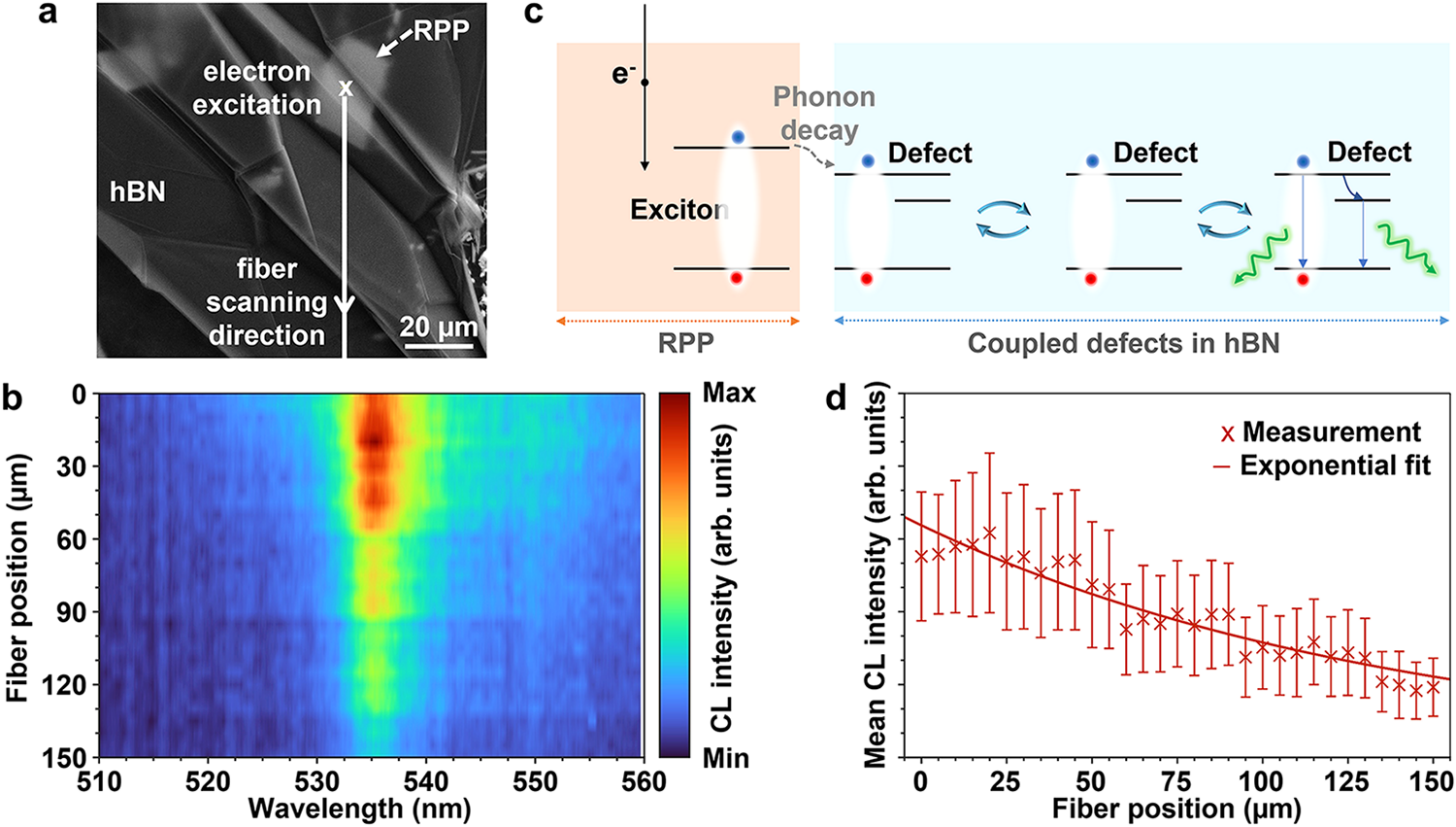
Long-range exciton transfer via exciton–defect Interactions. (a) Secondary-electron SEM image of the hBN/RPP sample, investigated by the fiber-based CL spectroscopy method. The electron beam incident point is fixed on the hBN/RPP heterostructure, shown by a white cross mark, while the fiber laterally scans the structure along the white arrow. (b) Measured CL spectra in the vicinity of the excitonic wavelength, collected along the scanning path, which exhibit the long-range exciton energy transfer through hBN over a distance of 150 μm away from the hBN/RPP heterostructure. (c) Proposed physical principle of the observed long-range exciton energy transfer through coupled defects in the hBN/RPP heterostructure. (d) Attenuation of the measured excitonic peak intensity versus the distance from the electron impact position, fitted to an exponential curve with a decaying coefficient of γ = 142.3 μm, which represents the exciton energy transfer length.

During this measurement, the electron beam position is fixed at the overlapping hBN/RPP heterostructure, marked by a white cross in Figure 4a. First, the focal point of the fiber is placed and aligned at the electron beam excitation spot, measuring the spectrum of the collected CL radiation around the excitonic wavelength of the hBN/RPP heterostructure, with a high spectral resolution. Next, the fiber’s focal point is laterally moved away from the fixed electron excitation spot, while measuring the CL spectra along the scanning path of the fiber (shown by the white line in Figure 4a).

The measured CL intensity (Figure 4b), originating from the exciton–defect coupling, remains significantly strong even at a long distance of 150 μm away from the excitation spot on the hBN/RPP heterostructure, assuring an ultralong-range exciton energy transfer through the hBN flake in the hBN/RPP heterostructure. This remarkably long-range excitonic energy transfer in addition to the long lifetime of excited excitons (Figure 1e) in the hBN/RPP heterostructure highlights unique aspects of hBN/2D perovskite heterostructures as an exciting class of optoelectronic materials in comparison with other platforms based on excitonic 2D materials at room temperature. Therefore, the hBN/RPP (*n* = 1) heterostructures offer an ultralong-range incoherent excitonic energy transfer, while benefiting from the inherently large exciton binding energy for RPP with *n* = 1. In other words, the hBN/RPP heterostructure can significantly elevate the inherent trade-off paradigm in 2D RPPs between the direct exciton transport range that is minimized for *n* = 1, growing by increasing *n*, and the exciton binding energy that is maximized for *n* = 1, decreasing for higher *n* values.^73^

### Mechanism of the Incoherent Excitonic Energy Transfer in the hBN/RPP Heterostructure

To explain the observed enhanced CL and the long-range excitonic energy transfer through the hBN/RPP heterostructure, here, we elucidate the mechanism of the coupling between excitons of RPPs and defects of hBN (Figure 4c). First, a moving high-energy electron (20 keV kinetic energy) excites an exciton in the underlying RPP flake, and the excited exciton couples to a defect center of about the same energy in the hBN flake via phonon decay. The excited defect further couples to the adjacent defect centers in the hBN layer via dipole-dipole interactions, partially transferring the excitonic energy through incoherent dipole–dipole coupling or defect–defect Förster energy transfer.^25,26,28,76^ Defects may interact as well through radiation-assisted energy transfer or photon recycling, where the radiation of one excited defect can be reabsorbed by the adjacent defect subsequently. Photon recycling, enhanced by the reflection of the photons from the boundaries of the layers, particularly can play a significant role in the radiation-assisted energy transfer.^33,34,77,78^

Defect–defect coupling, in addition to a sequence of photon recycling through defect emitters are believed to play significant roles in the observed ultralong-range incoherent excitonic energy transfer in the hBN/RPP heterostructure. The elucidated phonon-mediated exciton–defect coupling explains the previously observed slight red shift (about 5 nm) in the CL peak of the hBN/RPP heterostructure in comparison with the RPP excitonic peak (Figures 1d and 2e). Due to the presence of defect levels in hBN and their luminescence in this wavelength range (Figure 2f), the proposed exciton–defect–defect coupling can lead to radiative recombination at the red-shifted excitonic wavelength in the hBN/RPP heterostructure (a schematic of the many-body interactions is shown in Figure 4c). Furthermore, to quantify the effective length of this exciton energy transfer, we plot the measured excitonic CL peak intensity versus the scanning distance of the collecting fiber with respect to the electron impact position on the hBN/RPP heterostructure (Figure 4d), by fitting an exponential curve (α exp­(−*x*/γ)) to the acquired dataset, as shown in Figure 4d, where γ represents the effective exciton energy transfer length equal to 142.3 μm for the investigated hBN/RPP heterostructure. This observation confirms the efficient and ultralong-range exciton energy transfer through the coupled exciton–defect–defect system, originating from the inherently large density of point defects in hBN with an efficient Coulomb correlation and extensive hoping. This behavior is further confirmed by measurements performed on other investigated hBN/RPP heterostructures (see Figure 5 and Supporting Note 1 and Supporting Figure 1).

**Figure 5:**
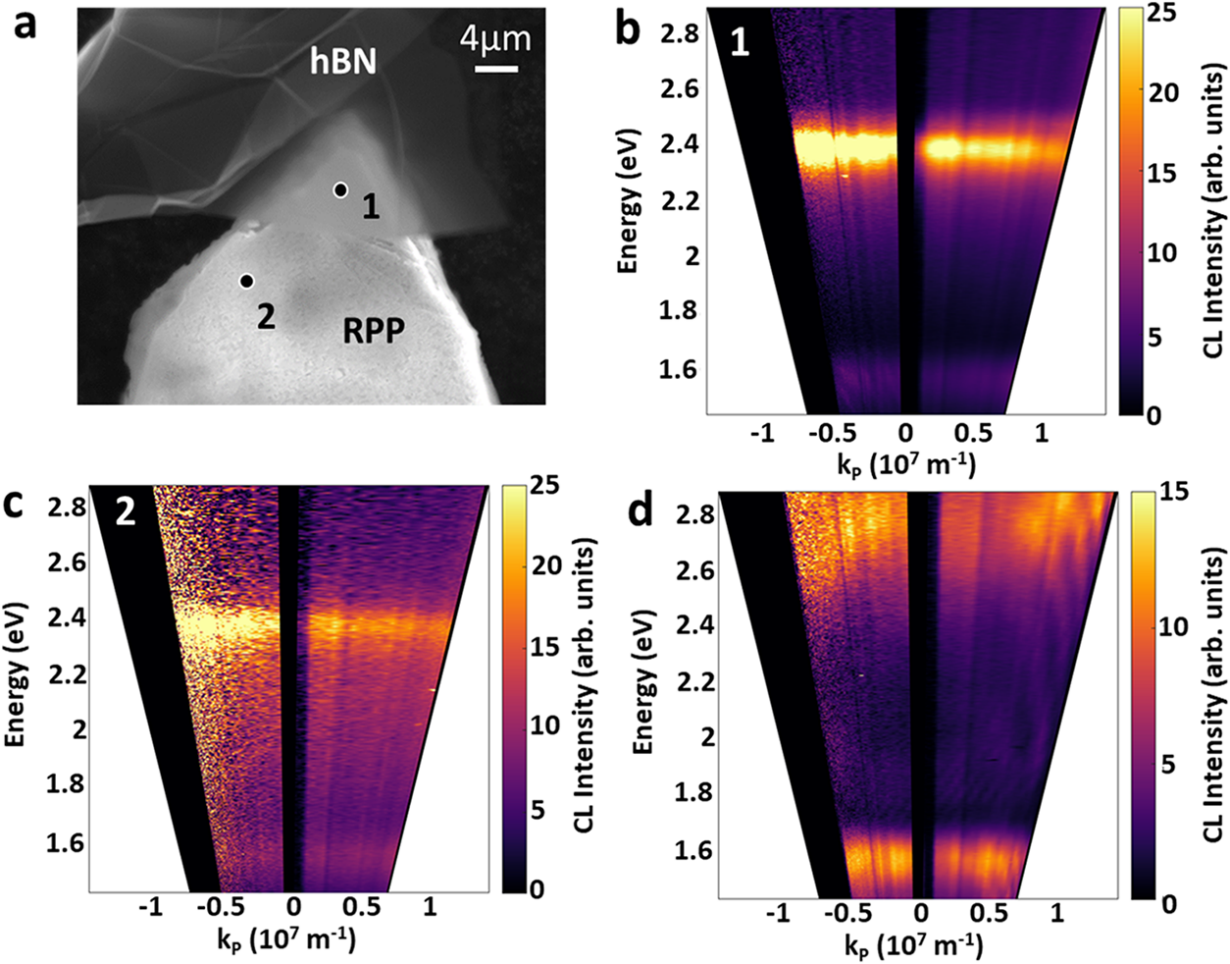
Energy-momentum map of the hBN/RPP heterostructures. (a) Secondary electron SEM image of the explored hBN/RPP structure. The energy-momentum map of the hBN/RPP heterostructures acquired at position 1 (b) and position 2 (c). The observed nearly flat band at the excitonic energy for the hBN/RPP heterostructure rejects polaritonic or waveguiding behavior and is in accordance with the proposed mechanism of coupled local defects. (d) Energy-momentum map of the pure hBN flake explored in Figure 2(f), provided for the sake of comparison. Electron beam current and acceleration voltage have been 200 pA and 20 kV in all parts, respectively.

To clarify the relative roles of photon-mediated (retarded dipole–dipole) coupling and purely near-field interactions, we developed a tight-binding model describing excitation transfer in a two-dimensional network of statistically distributed emitters (Supporting Note 6). The model incorporates distance-dependent dipolar coupling terms and simulates the dynamics of excitation hopping across the defect ensemble. The calculated spatial distribution exhibits an approximately exponential decay and a transport length that scales with emitter density and coupling strength, in agreement with the experimental observations. These results support a mechanism in which intermediate- and far-field radiative interactions dominate over purely near-field Förster-type processes in enabling long-range energy redistribution. The long-range energy transfer mechanism observed in the hBN/RPP heterostructure is not due to the coupling of the exciton emission to the waveguiding modes of hBN flakes. This claim is substantiated by the fact that no coherent response, such as the generally observed Fabry–Pérot interference patterns or interferences observed due to the interaction of waveguiding modes with the edges of the flakes are observed here in the CL hyperspectral images,^12,17^ but rather randomly positioned emission centers are confirmed. This effect is substantially explained in Supporting Note 7, where we compute the optical modes of the hBN/RPP/C heterostructure with hBN/C and pure hBN films and demonstrate experimental results where the waveguiding modes in free-standing hBN is confirmed, in contrast with hBN/C heterostructures. Moreover, using the mirror-collected CL spectroscopy, the energy-momentum maps of the emitted CL are measured on the hBN/RPP (*n* = 1) heterostructure and the RPP flake (labeled as ‘1′ and ‘2′ in Figure 5a), revealing nearly flat bands at the exciton energy of 2.4 eV (Figure 5b,c). The presented flat dispersion (Figure 5b) at exciton energy further validates a slow energy transfer mechanism as a result of exciton-assisted efficient excitation of coupled luminescent local defects in the hBN/RPP heterostructure, as opposed to the coupling of excitons with the waveguiding modes. The observed excitonic flat band aligns with the proposed energy transfer mechanism based on exciton–defect–defect coupling in the hBN/RPP heterostructure. For comparison, the energy-momentum map of the pure hBN flake is also provided (Figure 5d). All energy-momentum maps have been measured at the low current regime of 200 pA and an acceleration voltage of 20 kV.

To rule out the artifacts related to the utilized fiber collection geometry and secondary electron excitation, we conducted a control experiment using a pristine RPP flake without an hBN overlayer (see Supporting Figure 2). In this configuration, the electron beam with a low current (<200 pA) was fixed on the RPP flake, while the fiber collector was gradually displaced from the excitation point (Supporting Figure 2a). The resulting signal exhibited a short range that lies beyond the spatial resolution of our fiber-based CL detection. Here, the measured spatial decaying coefficient can be dominated by effects of the secondary electron excitation and the finite point spread function of the fiber with respect to the direct exciton transport range in pure RPP. Hence, the presented fiber-scanning CL measurement is not suitable for evaluating the short-range diffusion behavior, as opposed to the presented long-range energy transfer mechanism in the hBN/RPP heterostructure.

A mechanism contributing to the observed enhanced CL response in the hBN/RPP heterostructure can be the secondary or backscattered electrons from the underlying RPP flake that increase the emission yield from the defects in hBN. This behavior will be better clarified by the Monte Carlo simulations (see Supporting Note 2 and Figure 3). However, the major contribution is due to the multilevel defects in hBN and the exciton–defect–defect coupling in the hBN/RPP heterostructure, as discussed earlier and is solidified more in Supporting Figures 4 and 5. A further mechanism that could play a role in the observed CL response in the extruded hBN flake is the multiple and sequential elastic and inelastic scattering events of the excited secondary and backscattered electrons in the multilayer hBN/RPP structure. Generally, such mechanisms lead to a CL response from excitons, when the electron beam excites the hBN flake up to approximately several 100 nm away from the underlying RPP edge. As shown by the Monte Carlo simulations by considering the multilayered geometry, the CL response approaches zero after 2 μm away from the edge of the underlying RPP flake (see Supporting Note 2 and Figure 3). Therefore, this mechanism is only relevant at short distances away from the edge of the RPP flake and cannot substantiate the observed ultralong-range exciton energy transfer in the hBN/RPP heterostructure.

Degradation effects in RPP are due to different factors such as the environmental humidity and exposure to light or an electron beam.^43,50,51^ After one month of aging, the RPP flakes do not sustain a strong excitonic response, and the corresponding exciton–defect coupling and the enhanced CL peak at exciton energy are significantly faded, as well as the consequent long-range exciton energy transfer mechanism (see Supporting Information Note 3 and Figure 4). This observation clarifies the key role of excitons of RPPs and the resulting exciton–defect coupling in the observed enhanced and long-range CL behavior of the hBN/RPP heterostructure, ruling out a considerable contribution of the reflected electrons from the underlying RPP.

To elaborate more on the exciton–defect coupling in hBN/RPP heterostructures, hBN/RPP (*n* = 2) heterostructures are realized and probed by the mirror-collected CL spectroscopy (see Supporting Note 4 and Figure 5) at a beam current of 200 pA and acceleration voltage of 20 kV. The measured energy–distance CL intensity maps and the individual CL spectra at different positions of the hBN/RPP­(*n* = 2) heterostructure reveal a dominant CL peak at 580 nm and a weak peak at 530 nm on the hBN/RPP (*n* = 2) heterostructure. Considering the significant spectral overlap and the consequent coupling of the excitons in the pure RPP (*n* = 2)^73^ at around 590 nm with the phonon sideband peak of defects in pure hBN at 570 nm, this experiment strongly confirms the dominant role of RPP’s excitons in the emergence of the enhanced CL peak in the heterostructure, which is observed at about 580 nm in hBN/RPP (*n* = 2). Moreover, the weak spectral overlap of the RPP (*n* = 2) excitons with the interband transition of hBN’s defect states has also led to the emergence of a weak exciton–defect coupling peak at 530 nm in the hBN/RPP (*n* = 2) heterostructure. Therefore, it is proved that by changing the exciton energy of RPP in the hBN/RPP heterostructure, the energy of the coupled exciton–defect dominant peak is shifted from 525 nm for hBN/RPP (*n* = 1) to about 580 nm for hBN/RPP (*n* = 2). This experiment solidifies the mechanism of efficient exciton–defect coupling in hBN/RPP heterostructures, which can be tuned by changing *n* in RPP.

Next to the hBN/RPP (*n* = 2) structure and to better demonstrate that the defect–exciton coupling effect is a universal mechanism, we explored hBN/WS_2_ heterostructures as well. WS_2_ is a semiconducting TMDC supporting room-temperature A exciton at the wavelength of 610 nm, near the emission wavelength of RPP (*n* = 2). As shown in Supporting Information Note 9, the exciton spectral line partially overlaps with the second phonon sideband of hBN emitters. Hence, the coupling mechanism is not optimal and the energy transfer range of the excitons coupled to hBN emitters is significantly shorter than the RPP (*n* = 1)/hBN heterostructure.

CL spectroscopy is a major tool for exploring the coherent^12,79,80^ and incoherent^81^ dynamics of excitons in semiconductors. In addition, CL has been used to explore the photophysics of defects in hBN in various configurations.^48,56^ Compared to photoluminescence spectroscopy, where coherent and short-band light excitation interacts with samples, electron beams constitute a broadband excitation mechanism and can excite simultaneously excitons, phonons, and other classes of quasiparticle interactions, hence, more suitable to explore the interactions between quasiparticles in hybrid systems. Supporting Note 10 provides a comparison with photoluminescence spectroscopy performed on RPP and hBN/RPP structures.

Finally, we note that while radiative interactions between defects provide a suitable framework for describing the long-range exciton energy transfer observed here, additional effects, such as photon recycling and partial reflections at boundaries, as well as strong interactions of defects with phonon polaritons, may also influence the effective interaction strength. A hybrid description incorporating these contributions may therefore offer an even more complete picture of the system. Importantly, conventional coherent waveguiding mechanisms cannot account for the experimental observations. Although an extremely high defect density could, in principle, lead to an effective Lorentzian dielectric response with strong oscillator strength and the formation of surface waves, such a regime would correspond to defect clustering and would result in significantly altered luminescence characteristics, which are not observed here. Moreover, coherent guided modes would give rise to distinct interference patterns, as discussed in the Supporting Information, which are absent in our measurements. Therefore, we conclude that radiative interactions within a dense, disordered defect network provide the most consistent explanation for the observed exponential decay, the dependence of transport length on defect density, and the spatially incoherent nature of the energy redistribution.

## Conclusions

Benefiting from a high exciton binding energy at room temperature, RPPs also exhibit a slow exciton annihilation rate and relatively long exciton diffusion lengths, compared to their TMDC counterparts. The combination of these extraordinary optical properties in addition to their simple synthesis and composition tunability entitles 2D RPPs as superior candidates for optoelectronic applications, while their inherent degradation in exposure to light and electron beams have remained as their main drawback.

Here, we have explored hBN/RPP heterostructures by CL spectroscopy, which was allowed by the surpassed electron beam-induced degradation of RPPs at the low-current regime. The hBN/RPP (*n* = 1) heterostructure revealed enhanced luminescence intensity, narrowed emission bandwidth, increased decay time of the excited excitons, and a slight red shift in the excitonic wavelength of RPPs. Furthermore, benefiting from the dual scanning functionality of a unique fiber-collected CL spectroscopy setup, an ultralong-range exciton energy transfer length of about 150 μm was proved through hBN in the hBN/RPP (*n* = 1) heterostructure. The observed enhanced CL spectrum and the ultralong-range exciton energy transfer were attributed to the exciton–defect coupling in the hBN/RPP heterostructure and the defect–defect coupling in addition to the photon recycling through the defect emitters in hBN. Realizing high-quality hBN/RPP (*n* = 1) heterostructures intensively enhanced the exciton energy transfer, while keeping the benefit of the inherently highest exciton binding energy with respect to other RPPs with *n* > 1, emphasizing the potential of hBN/RPP (*n* = 1) heterostructures for applications in active optoelectronics and light-harvesting devices.

## Methods

### Sample Preparation

To achieve 2D RPP (*n* = 1) bulk crystals,^1^ initially, *n*-butylammonium iodide (BAI) is synthesized by gradually adding 25 mL of 57% w/w aqueous hydriodic acid (HI) to 5 mL of *n*-butylamine (BA) within an ice bath, while stirring for 4 h. Next, 500 mg of PbO powder is dissolved in a mixture of HI (57%,3 mL) and H_3_PO_2_ (50%,850 μL) at a temperature of 120 °C, while stirring the solution for about 5 min until converting it into a bright-yellow solution of PbI_2_. Thereafter, 1.5 mL of the synthesized *n*-CH_3_(CH_2_)_3_NH_3_I (BAI) is added to the prepared PbI_2_ solution, resulting in a transient black precipitation that is dissolved by keeping the solution temperature at 120 °C. After 5 min in this condition, stirring and heating are stopped, letting the solution to cool to room temperature, during which the orange bulk crystalline BA_2_PbI_4_ emerged among the solution. Using vacuum filtering, BA_2_PbI_4_ products are separated and dried for a few days.

Next, to realize the van der Waals hBN/RPP heterostructure, the synthesized BA_2_PbI_4_ crystalline flakes have been mechanically exfoliated onto a double-sided adhesive carbon tape. For this purpose, a BA_2_PbI_4_ flake was picked up by a piece of scotch tape, and after folding and unfolding several times, several flakes with reduced thicknesses were achieved, which were stamped on the carbon tape subsequently. HBN crystals were purchased from the HQ Graphene Company. Utilizing mechanical exfoliation, thinned hBN flakes were transferred on the RPP flakes that were previously transferred on the carbon tape. Overlapping of the large hBN flakes on the underlying RPP flakes and van der Waals hBN/RPP heterostructures are achieved.

### Degradation Issue in Cathodoluminescence Spectroscopy of hBN/RPP Heterostructures: Electron Beam-Induced Degradation in RPPs

The presence of an hBN flake on top of RPP significantly protects the RPP from electron beam-induced degradation. In addition, to investigate the suitable measurement conditions for CL spectroscopy on RPP flakes, we systematically investigated the electron beam conditions to find optimum settings. Exposing partially covered RPP flakes by hBN to high-current electron beams reveals drastic degradation in the uncovered parts of the RPP flake. In contrast, the hBN cover results in efficient protection against electron beam irradiation (Supporting Information Note 5 and Figure 6). Moreover, an acceleration voltage of 20 kV and a low-current regime of about 200 pA are best used to efficiently excite excitons in RPP flakes, while avoiding the electron beam-induced degradation, even without hBN protection (Supporting Information Figure 7).

### Fiber-Collected Cathodoluminescence Spectroscopy and Time-Correlated Single-Photon Counting

The measurements shown in Figures 1, 4, S1, and S2 were performed by using a Thermo Fisher Quattro S field-emission scanning electron microscope (FE-SEM). Throughout the experiments, the acceleration voltage and the current of the electron beam were adjusted at 20 kV and 140 pA. A built-in amperemeter is connected to the sample stage in the SEM, measuring the effective beam current at the investigated specimen. Inside the SEM, a system of three perpendicular linear piezo-driven stages from SmarAct is mounted, holding an optical multimode fiber, which can be moved independently from the sample stage along three axial degrees of freedom. The stages feature dynamic ranges of 83 mm along the *x*-axis and 35 mm along the *y*- and *z*-axis with an accuracy of 1 nm in step size, allowing for a precise and high-spatial selectivity in the collection of the cathodoluminescence radiation by the fiber. For the experiments, we used the optical multimode fiber FG400AEA from Thorlabs, which has a silica core diameter of (400 ± 8) μm with a broad spectral operation bandwidth from 180 nm up to 1200 nm and a numerical aperture of 0.22 ± 0.02 for the flat fiber cross section. To improve the numerical aperture and therefore the collection efficiency, a lens was 3D-printed onto the face of the multimode fiber, using the Quantum X 3D printer from Nanoscribe GmbH.^82–85^ The numerical aperture of the fiber with this micro-optic has been increased to 0.36 with a working distance of 400 μm approximately, while the fiber has a fixed inclination angle of 35° with respect to the sample stage. The electron beam position and its spot size are controlled via the electron optics of the SEM. The presented fiber-collected CL configuration allows for adjusting the position of the focal point of the fiber with respect to the electron beam impact position on the sample. The lateral (in-plane) position of the fiber is measured by using the secondary electron detector of the SEM apparatus, whereas the vertical (out-of-plane) position of the fiber is fixed at the position with the highest collected CL intensity from the investigated sample.

The fiber cable guides the collected CL radiation toward the detectors, either a spectrometer or photomultiplier tubes. For the spectroscopy measurements, we use a Teledyne Princeton Instruments HRS-500 spectrograph attached with a PyLoN 100BRX liquid nitrogen-cooled CCD camera. It is equipped with three gratings, all of which have a groove density of 1200 mm^–1^ with a spectral resolution of 0.1 nm and blaze wavelengths of 300, 500, and 750 nm, individually. In addition, a Hanbury Brown–Twiss intensity interferometer is utilized for performing time-correlated single-photon counting and measuring the second-order autocorrelation function *g*_CL_^(2)^(τ) of the emitted CL intensity signal. This assembly consists of two PMA Hybrid 50 photomultiplier tubes from PicoQuant, the quTAG time-to-digital converter from qutools, and the TT400R5F1B 1 × 2 multimode fiber coupler from Thorlabs with 50:50 splitting ratio. The output signal of the photomultiplier tubes has a pulse duration of approximately 1.2 ns (FWHM). However, the achievable time resolution for this setup is mostly determined by the leading edge’s steepness of 300 ps and the transit time spread of less than 160 ps, resulting in an error in the range of 0.3 to 0.4 ns. Furthermore, the time-to-digital converter has an input timing jitter of less than 25 ps (FWHM) and a delay resolution of 1 ps, which refers to the smallest available bin width.

### Mirror-Collected Cathodoluminescence Spectroscopy

As a complementary measurement, we also performed CL spectroscopy using an aluminum-coated parabolic mirror to collect the emitted CL radiation, shown in Figures 2, 3, 5, and S4–S7. These measurements were performed inside a ZEISS Sigma field-emission scanning electron microscope, equipped with the Delmic SPARC CL system. Throughout these measurements, the acceleration voltage of the electron beam was set to 20 kV, whereas the beam current was kept at 200 pA to hinder electron beam-induced degradation in RPPs, unless otherwise specified. The CL detector consists of an off-axis paraboloid mirror, which is positioned with an accuracy less of than 1 μm above the specimen and redirects the collected CL radiation onto the analyzing path and a CCD camera (Andor i-KonM) for further analysis. The parabolic mirror has an acceptance angle of 1.49π sr (NA = 0.97) and a focal distance of 0.5 mm. The exciting electron beam can pass the mirror through a hole with a diameter of 600 μm located above the focal point. Utilizing a dispersive reflection grating and on occasion directing the CL radiation through a one-dimensional slit opening, the CL detector is capable of performing hyperspectral and energy-momentum imaging. The acquisition time for each pixel was set to 200 ms for hyperspectral imaging and 120 s for energy momentum mapping.

## Supplementary Material



## Data Availability

The dataset generated during and/or analyzed during the current study are available from the corresponding author upon reasonable request.

## Notes

Complementary fiber-collecting CL measurements to confirm the long-range energy transfer in hBN/RPP (*n* = 1) heterostructures, Monte Carlo simulations of the scattered electrons in the hBN/RPP heterostructure effect of RPP degradation on the optical response of the hBN/RPP heterostructure, exploring the hBN/RPP (*n* = 2) heterostructure by mirror-collecting cathodoluminescence, exploring the electron beam-induced degradation in RPPs, tight-binding model for defect–defect interactions and photon-mediated defect networks, waveguiding behavior of hBN/RPP/C and hBN/C heterostructures, fitting procedure for the fiber-assisted CL spectroscopy measurements, investigation of a complementary WS_2_/hBN/C heterostructure via mirror-collected cathodoluminescence, and complementary photoluminescence measurements of RPP/C and RPP/hBN/C heterostructures (PDF)

## Notes

The numerical code used to simulate the data are available from the corresponding author upon reasonable request.
