# Supplementary material for: Exciton Energy Routing via Defect Networks in hBN/2D Perovskite Hybrids
Source: ACS Nano. 2026 Jul 25;20(31):21739–51. doi: 10.1021/acsnano.6c03445 (PMC13472323; doi:10.1021/acsnano.6c03445)
Supplement: Supplementary file 1 [file nn6c03445_si_001.pdf]

Supporting Information

Exciton Energy Routing via Defect Networks in hBN/2D-Perovskite Hybrids

Sara Darbari[1,2,†*], Paul Bittorf[1,†], Leon Multerer[1], Fatemeh Chahshouri[1], Parsa Darman[1,2], Pavel Ruchka[3], Harald Giessen[3], Masoud Taleb[1,4], Yaser Abdi[1,5], Nahid Talebi[1,4*]

1 Institute of Experimental and Applied Physics, Kiel University, 24418 Kiel, Germany

2 Faculty of Electrical and Computer Engineering, Tarbiat Modares University, Tehran 1411713116, Iran

3 4th Physics Institute and Research Center SCoPE, University of Stuttgart, 70569 Stuttgart, Germany

4 Kiel Nano, Surface, and Interface Science KiNSIS, Kiel University, 24118 Kiel, Germany

5 Department of Physics, University of Tehran, Tehran 1439955961, Iran

† These authors contributed equally to this work.

Corresponding Authors:

**Email*: talebi@physik.uni-kiel.de

**Email*: s.darbari@modares.ac.ir

Supplementary Note 1: Complementary fiber-collecting cathodoluminescence measurements to confirm the long-range energy transfer in hBN/RPP(*n*=1) heterostructures.

Supplementary Note 2: Monte Carlo Simulations.
Scattered electrons in the hBN/RPP heterostructure.

Supplementary Note 3: The effect of RPP degradation on the optical response of the hBN/RPP heterostructure.

Supplementary Note 4: Exploring the hBN/RPP(*n*=2) heterostructure by mirror-collecting cathodoluminescence.

Supplementary Note 5: Exploring the electron-beam-induced degradation in RPPs.

Supplementary Note 6: Tight-binding model for defect–defect interactions and photon-mediated defect networks

Supplementary Note 7: Waveguiding behavior of hBN/RPP/C and hBN/C heterostructures

Supplementary Note 8: Fitting procedure for the fiber-assisted CL spectroscopy measurements

Supplementary Note 9: Investigation of a complementary WS_2/hBN/C heterostructure via mirror-collected cathodoluminescence

Supplementary Note 10: Complementary photoluminescence measurements of RPP/C and RPP/hBN/C heterostructures

Supplementary Note 1: Complementary fiber-collecting cathodoluminescence measurements to confirm the long-range energy transfer in hBN/RPP(*n*=1) heterostructures

The optical properties of another RPP flake that was partially covered by a large hBN flake is studied here by utilizing the fiber-collected CL spectroscopy. First, we scan the sample by moving the electron-beam excitation spot while keeping the position of the detecting fiber fixed (at the cross mark in Supplementary Figure 1a). Second, the detecting fiber is moved over the heterostructure, while keeping the electron excitation position fixed (at the cross mark in Supplementary Figure 1d). Thereby, we would be able to determine the effect of changing the excitation and detection positions in the investigated hBN/RPP heterostructure on the CL emission, in addition to probing the propagation characteristic of the excited excitons at the hBN/RPP heterostructure over long-range distances. In the first measurement, when the electron-beam impacts the tapered-like edge of the unencapsulated RPP flake, edge excitons are efficiently excited, leading to a strong and broad CL peak (shown by orange zones and spectrum in Supplementary Figure 1(a-c)). However, when the electron-beam impacts the surface of the unencapsulated RPP flake the excitonic peak intensity decreases and remains constant, until the electron-beam impacts the hBN edge on top of RPP flake. Scanning to the hBN/RPP heterostructure by passing through the hBN edge (shown by red zones and spectrum in Supplementary Figure 1(a-c)), the excitonic peak increases in consistency with the luminescent enhancement in Fig. 1d and Fig. 2c, d, e. Next, passing over the underlying RPP edge and scanning on the extruded part of hBN (shown by black zones and spectrum in Supplementary Figure 1(a-c)) the excitonic peak intensity is decreased, but remains at a constant value. Scanning the electron-beam over the wrinkles in the extruded hBN flake leads to a higher CL intensity at the excitonic peak, which can be attributed to exciting higher local defects density in hBN that improves the resonance response of the coupled exciton-defect system. Moreover, the emergence of the narrowed, red-shifted CL spectra in exciting the hBN/RPP heterostructure (red peak), and the extruded part of hBN (black peak) are reconfirmed in Supplementary Figure 1(c).

In the second step of this measurement, by scanning the fiber away from the RPP edge, while continuously measuring the emitted CL spectra, we observe the excitonic peak at 535 nm on the extruded part of the hBN flake over a long distance up to 100 μm (Supplementary Figure 1(d, e)). Moreover, a higher excitonic CL intensity is collected at hBN wrinkles, which is related to higher portion of excitonic radiative decay at defects, discontinuities and edges (Supplementary Figure 1(e)). This measurement confirms a spatial exponentially decaying behavior of the excitonic peak intensity over the long-range, wherein an exponential curve with decaying coefficient of $\gamma = 154.9\ \mu m$ is fitted to the average measured CL intensities (Supplementary Figure 1(f)), comparable with the measurement in Fig. 4d.

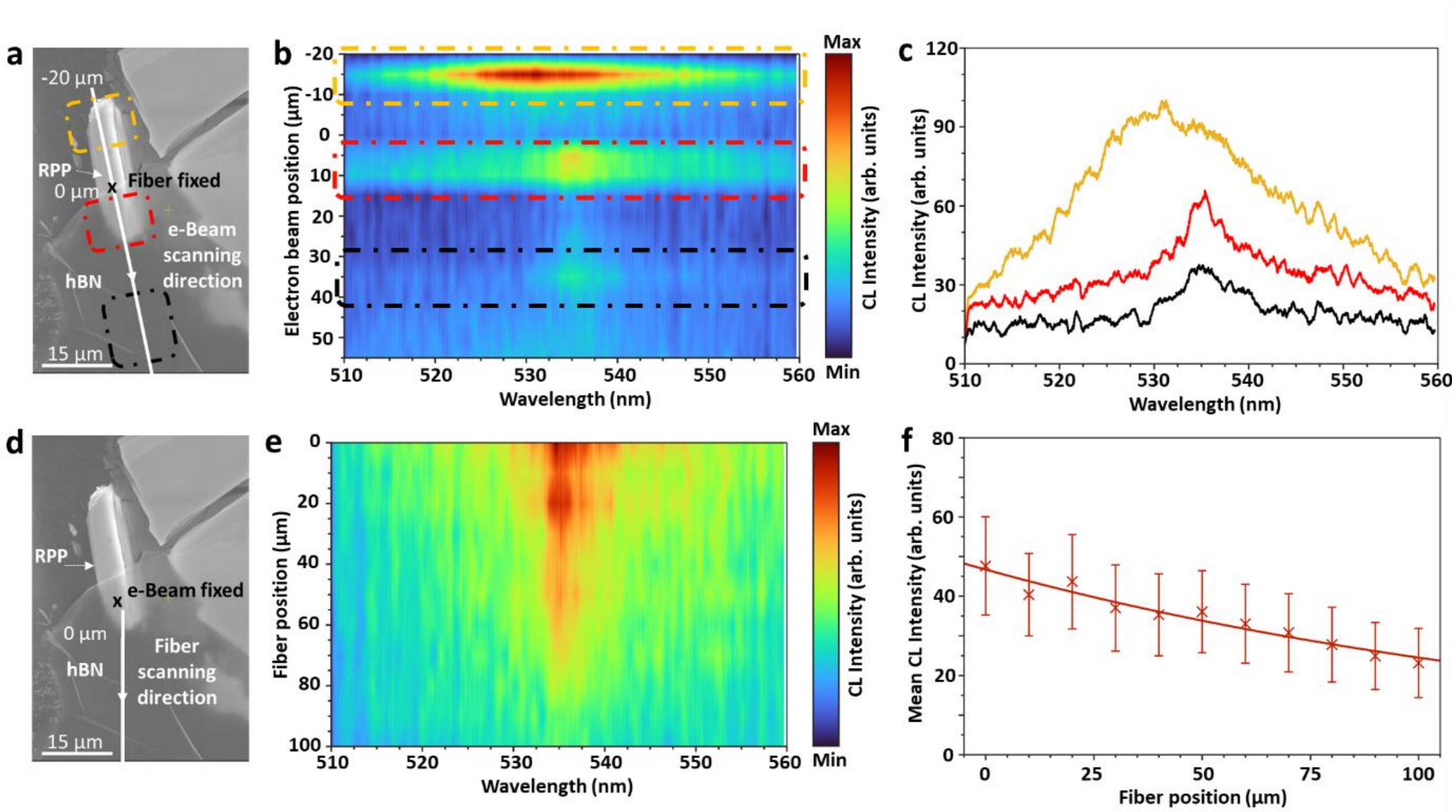

Supplementary Figure 1: Fiber-collected CL spectroscopy results of the hBN/RPP heterostructure. Secondary electron SEM image of the investigated RPP structure is demonstrated in both (a) and (d). (a) Fiber position is fixed on the sample at the cross mark, while electron-beam is scanning the structure along the white arrow. (b) The measured CL spectra along the electron-beam scanning path. The regions highlighted by the dash-dotted boxes correspond to the regions marked on the SEM image with the same colors. (c) CL spectra integrated over the selected regions specified by colored boxes in (b). (d) The electron-beam position is fixed on the hBN/RPP heterostructure at the cross mark, while the fiber is scanning along the white arrow. (e) The measured CL spectra along the fiber's scanning path in part (c). (f) The mean excitonic peak intensity and standard deviation of the measured CL spectra from part (e) versus the scanning fiber position, fitted to an exponential function of exp(-x/γ) with a decaying coefficient of γ=154.9 μm.

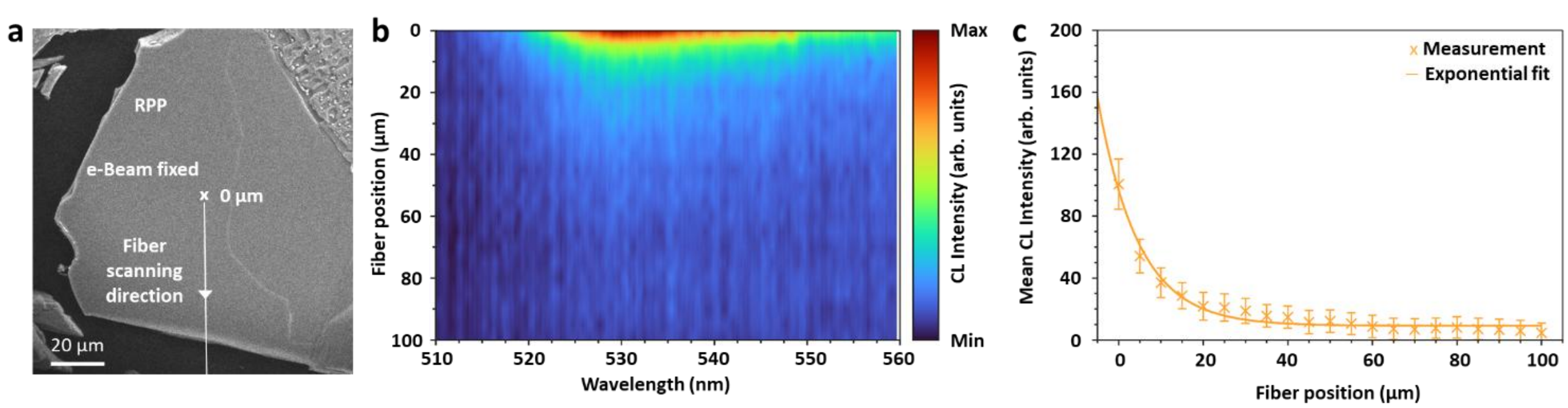

Supplementary Figure 2 | Fiber-collected cathodoluminescence (CL) spectroscopy of a pristine RPP flake. (a) Secondary electron SEM image of the investigated RPP flake. The electron-beam is fixed at the position marked by the cross, while the fiber is scanned along the direction indicated by the white arrow. (b) CL spectra measured along the fiber scanning path shown in (a). (c) Mean peak intensity (with standard deviation) of the CL spectra from (b) plotted as a function of fiber position. The data are fitted to an exponential decay function of the form exp(–x/γ), yielding a decay length of γ = 9.6 μm.

Spatial decaying behavior of the excitonic CL peak is also measured on a large pure RPP flake without any hBN flake. As illustrated in Supplementary Figure 2(a), electron-beam is fixed at the cross mark to excite RPP's excitons, and the CL-collecting fiber is scanning along the white arrow, up to 100 μm away from the excitation point. Supplementary Figure 2(b, c) exhibit the collected excitonic CL peak and the mean CL peak intensity versus the fiber position, revealing the fast-decaying behavior of the peak intensity in pure RPP along a few micrometers in contrast with the hBN/RPP heterostructure.

Supplementary Note 2: Monte Carlo Simulations.

In order to investigate the contribution of the secondary electrons and backscattered electrons of individual layers to the CL response, we perform depth-resolved electron energy-loss simulations using CASINO Software[1-3]. This approach has been proven to efficiently reproduce CL responses beyond Eikonal approximations, when combined with Maxwell's equations[4]. In this package, the elastic scattering events have been calculated using the tabulated Mott cross-sections, and the inelastic interactions have been approximated by the mean energy loss rate between the successive collisions, as described by the modified Bethe law by Joy and Luo[1]. The simulations employ a single-scattering model to track the electron trajectories and compute energy loss, expressed as $\Delta E = \frac{dE}{dx}.d$, where $\frac{dE}{dx}$ is the mean energy loss rate, and d is the distance between collisions[1,2]. Trajectory calculations terminate when electrons exit the sample, or their energy falls below a threshold value of 50 eV. For each scan point, the total deposited energy is calculated by summing energy loss contributions across the volume to generate energy-loss maps, revealing spatial distributions of energy absorption and scattering in the hBN/RPP heterostructures (Supplementary Figure 3a to c). The energy deposition can also be analyzed as linear scans or cross-sectional views, providing insights into dynamic or static beam-sample interactions (Supplementary Figure 3d to f).

The electron-beam is assumed to have a beam diameter of 10 nm, primary beam energy of 20 kV, and an incident electron number of 200,000. Cross-sectional views of energy-loss maps as a function of the electron-beam position on a hBN/RPP heterostructure show the amount of the absorbed energy within the sample, scaling from black (no absorbed energy) to white (maximum absorbed energy) in Supplementary Figure 3a to c. As the penetrating electrons interact with the layers, they undergo both elastic and inelastic scatterings, resulting in their lateral and vertical trajectories as well as the generation of secondary electrons. When the electron-beam impinges on the sample, 2000 nm away (on the left side) from the vacuum/RPP boundary, the electrons undergo significant energy loss in the top hBN layer and in the bottom carbon substrate (Supplementary Figure 3a).

For the case of electron-beam impinging directly at the RPP edge, the electrons that penetrate the hBN/RPP region experience significant inelastic scatterings due to the underlying RPP layer in the right half (Supplementary Figure 3b). When the electron-beam impinges 2000 nm away (right side) from the underlying vacuum/RPP boundary, a huge energy loss inside the RPP layer is observed

(Supplementary Figure 3c). Better clarified by the linear scans, the maximum percentage of electron energy-loss occurs at a depth of approximately 3 µm in the bottom carbon tape when electron-beam impinges the extruded part of the hBN layer (Supplementary Figure 3d), whereas the majority of energy dissipation occurs within the RPP layer at a depth of approximately 1 µm for excitation on the hBN/RPP heterostructure (Supplementary Figure 3e). Moreover, a higher portion of electron energy loss occurs in the hBN layer when the underlying RPP presents below it in the hBN/RPP heterostructure, which is due to the scattered electrons from the RPP back into the hBN. As a clear confirmation, 2D simulation of the electron trajectories within the hBN/RPP heterostructure is presented in the inset of Supplementary Figure 3e, where red trajectories represent backscattered electrons and blue trajectories indicate absorbed electrons. Additionally, the fraction of secondary electrons captured in the RPP layer as a function of the electron-beam impinging x-position is plotted in Supplementary Figure 3f, wherein the electron-beam is scanned along x-axis over the RPP edge (at *x*=0 nm). As observed, the fraction of captured secondary electron in RPP that are responsible for the observed excitonic CL peak, increases with a sharp step-like transition at the RPP edge. Therefore, the simulated short-range trajectory of secondary electrons in the extruded part of the hBN layer (Supplementary Figure 3f) confirms that the observed CL signal, when the electron-beam impacts the structure at locations far from the RPP flake, cannot be attributed to the excitation of RPP excitons by backscattered electrons.

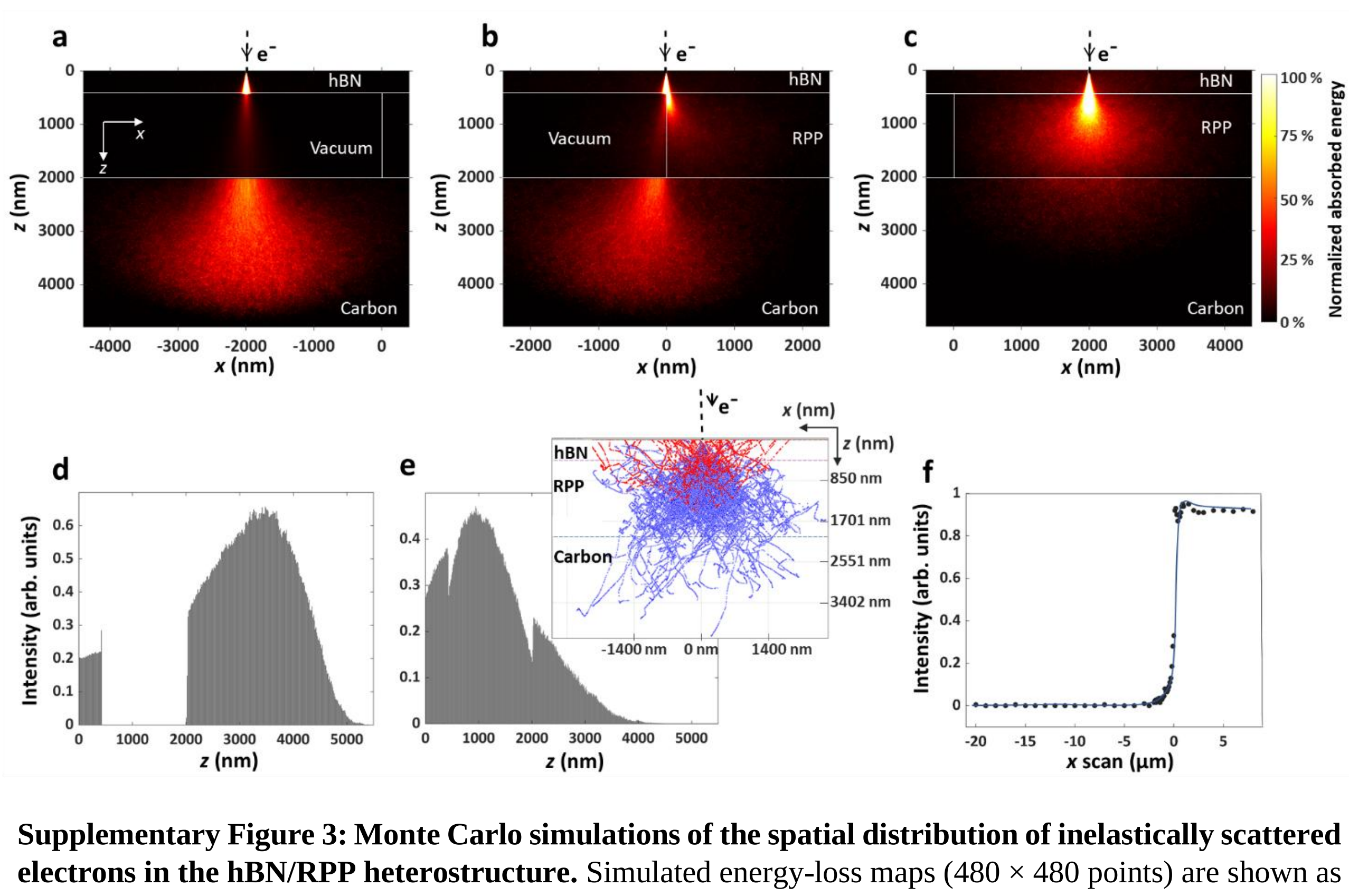

Supplementary Figure 3: Monte Carlo simulations of the spatial distribution of inelastically scattered electrons in the hBN/RPP heterostructure. Simulated energy-loss maps (480 × 480 points) are shown as a function of electron-beam scanning position on (a) a suspended hBN film over a carbon substrate at the distance of 2000 nm away from the RPP/vacuum interface, (b) the vacuum/RPP interface positioned on a carbon substrate, and (c) the hBN/RPP heterostructure at the distance of 2000 nm away from vacuum edge. Each map is generated with 200,000 incident electrons, and a beam diameter of 10 nm. Simulated electron

energy-loss profiles for (d) the hBN/Vacuum/Carbon structure and (e) the hBN/RPP/Carbon structure. The inset illustrates electron trajectories of the secondary electrons (blue) and backscattered electrons (red) generated by a 20 kV electron-beam. (f) Simulated secondary-electron fraction captured in RPP as a function of electron-beam position from the RPP/vacuum edge.

Supplementary Note 3: The effect of RPP degradation on the optical response of the hBN/RPP heterostructure

The effect of RPP's inherent degradation on the CL characteristic of the hBN/RPP heterostructure is investigated by the mirror-collected CL spectroscopy technique. The secondary electron SEM image and the measured CL spectra of the freshly prepared hBN/RPP heterostructure are presented in Supplementary Figure 4a and b, and comparing it with the CL spectra of the same heterostructure after 30 days in Supplementary Figure 4c and d. The CL measurements are collected during moving the electron-beam excitation spot along the blue arrows in parts a and c, at low current regime of 200 pA and acceleration voltage of 20 kV. The degraded underlying RPP experiences from a more significant electron charging effect and a reduced quality in the achieved secondary electron SEM image in Supplementary Figure 4c, as compared with Supplementary Figure 4a. Comparing the measured CL spectra in Supplementary Figure 4b and d reveals that the enhanced peak at 525 nm on the fresh hBN/RPP heterostructure diminishes to a very weak trace of excitonic peak in the degraded sample. This evidence solidifies the proposed exciton-defect coupling in hBN/RPP heterostructure as the main origin of the observed enhanced CL at 525 nm before degradation of RPP, as opposed to other effects such as contribution of the secondary electrons and backscattered electrons of individual layers, such as the bottom RPP layer, to the CL response.

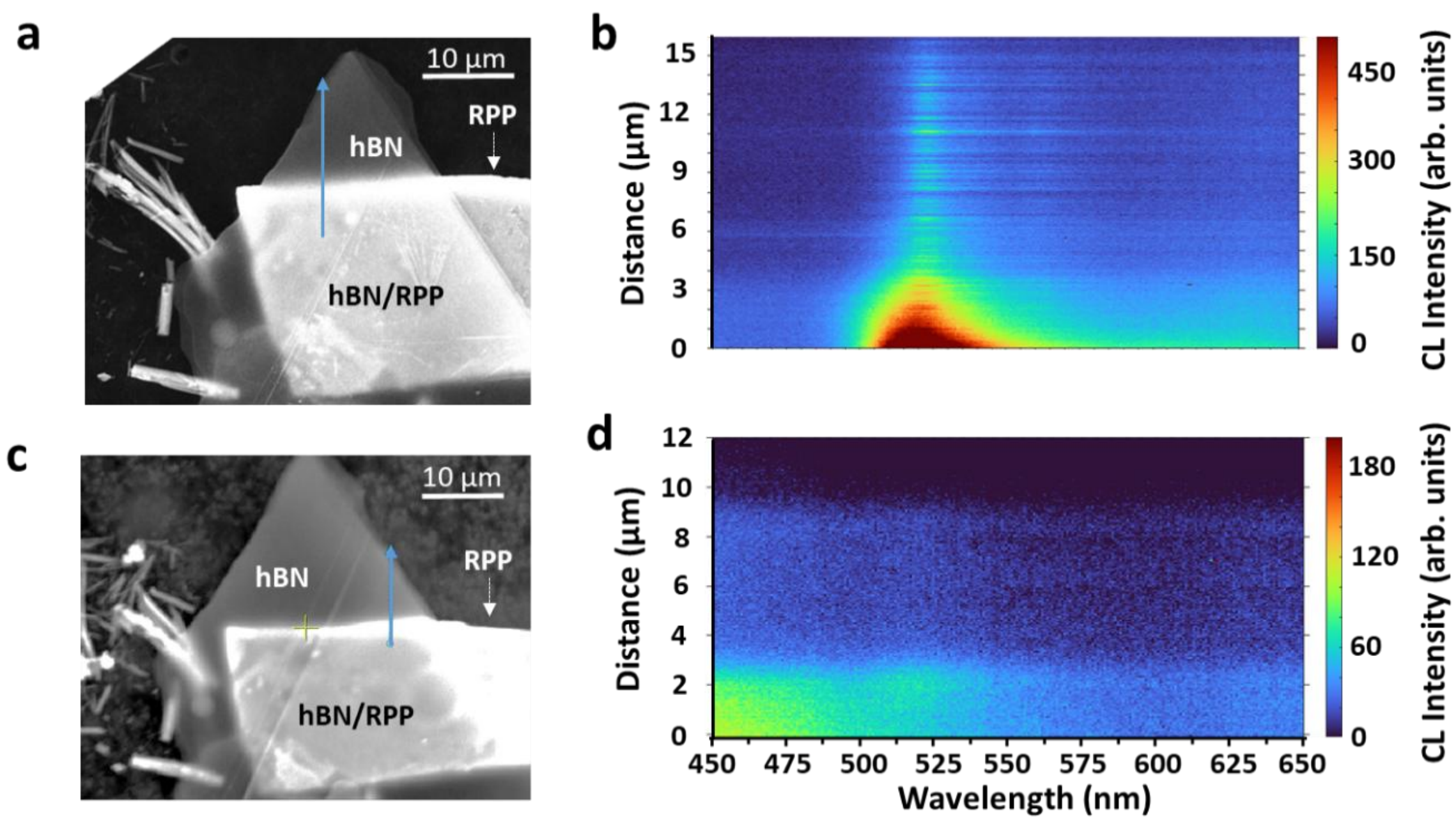

Supplementary Figure 4: Effect of one-month aging in RPP explored by the mirror-collected CL system. SEM image of (a) the prepared fresh hBN/RPP heterostructure, and (c) the same structure after one month, wherein blue arrows show the electron-beam scanning length. The measured CL spectra along the electron-beam scanning length (b) for the fresh hBN/RPP sample, and (d) for the same hBN/RPP sample after one month.

Supplementary Note 4: Exploring the hBN/RPP(n=2) heterostructure by mirror-collecting cathodoluminescence.

To elaborate more on the exciton-defect coupling in hBN/RPP heterostructure, hBN/RPP(*n*=2) heterostructures are realized and probed by the mirror-collected CL spectroscopy setup (Supplementary Figure 5). In this CL measurement, the electron-beam with 200pA current is moved along the blue arrow on the hBN/RPP(*n*=2) (part a), passing over the underlying RPP edge, and collecting the emitted photons. The measured energy-distance CL intensity maps and the individual CL spectra at different positions marked by ‘1’ to ‘4’ in part (a) reveal a dominant CL peak at 580 nm and a weak peak at 530 nm on the hBN/RPP(*n*=2) heterostructure (marked by ‘4’). Moreover, the dominant peak intensity is drastically decreased by sweeping the electron-beam toward the extruded part of the hBN away from the underlying edge of RPP (marked by ‘1’). However, the weak peak at 530 nm is still observable with a comparable intensity on the extruded hBN at position marked by ‘1’. To clarify the exciton-defect coupling in the hBN/RPP(*n*=2) heterostructure, the CL spectrum of a pure RPP(*n*=2) is superimposed on the CL trace of a pure hBN flake, acquired at a high beam current of 7 nA (part d). This experiment strongly proves the

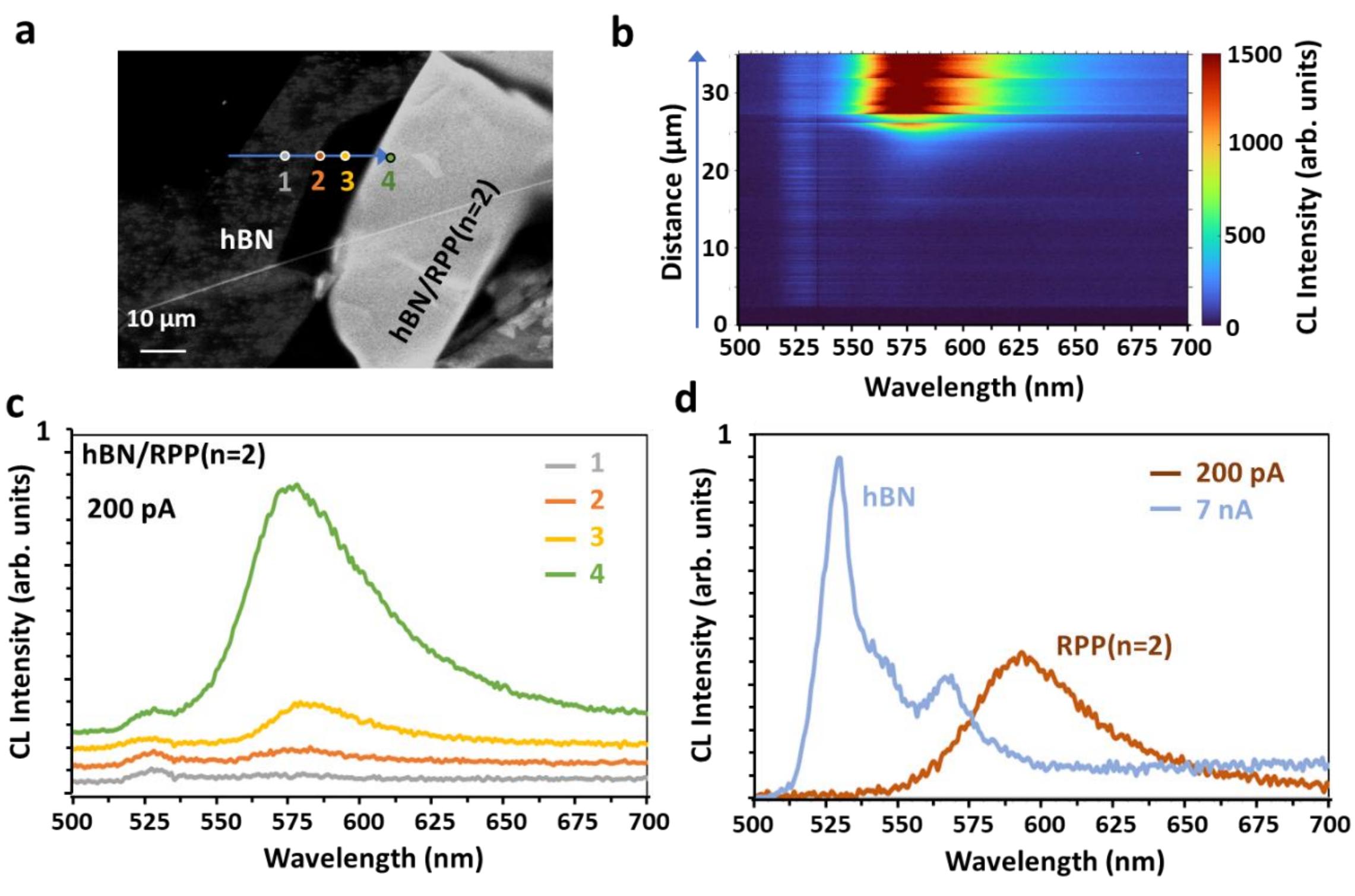

Supplementary Figure 5: Spatially-resolved cathodoluminescence response of the hBN/RPP(*n*=2) heterostructure. The exciton-defect coupling in hBN/RPP(*n*=2) has been studied the mirror-collected CL spectroscopy setup at the beam current of 200 pA and acceleration voltage of 20 kV (a) Secondary-electron SEM image of the hBN/RPP(*n*=2), wherein the blue arrow shows the electron-beam scanning path across the underlying RPP(*n*=2) edge. (b) The measured CL spectra along the blue arrow in part (a). (c) Individual CL spectra corresponding to four different spots, marked as ‘1’, ‘2’, ‘3’ and ‘4’ in part (a). CL spectra of a pure hBN flake (without RPP) integrated over a large area at the low current (200 pA) and high current (7 nA) regimes, superimposed on the CL spectrum of a pure RPP(*n*=2) flake at low current (200 pA) regime.

dominant role of exciton wavelength in emergence of the enhanced CL peak due to the coupled exciton-defect state at the hBN/RPP heterostructure. Here, excitons of RPPs(n=2) at 590 nm show significant spectral overlap with the phonon sidebands of hBN defects at 570 nm, and negligible spectral overlap with the defect peak at 530 nm (Supplementary Figure 5d), leading to emergence of a dominant peak at about 580 nm and a weak peak at around 530 nm on the hBN/RPP(n=2) heterostructure. Thus, by changing the exciton energy in the underlying RPP by changing the n value during the chemical synthesis step, we can tune the energy of the coupled exciton-defect states in the hBN/RPP heterostructures.

Supplementary Note 5: Exploring the electron-beam-induced degradation in RPPs

To investigate the suitable conditions for CL measurements on RPP-based samples, we utilize two approaches: (i) benefiting from hBN-encapsulation in the hBN/RPP heterostructure, and (ii) finding the appropriate electron-beam conditions to achieve an efficient exciton excitation and avoiding the degradation issue in RPPs.

To evaluate the protection functionality of the hBN layer against electron-beam irradiation, RPP flakes that are partially covered by a large hBN flake (Supplementary Figure 6a) are exposed to an intense electron-beam condition at high sample current of 16 nA and acceleration voltage of 10 kV in the scanning electron microscope (SEM). The uncovered part of RPP flakes show a drastic electron-beam-induced degradation, while the covered part is protected (Supplementary Figure 6b). Therefore, the realized hBN/RPP heterostructure surpasses the electron-beam-induced degradation in CL measurements.

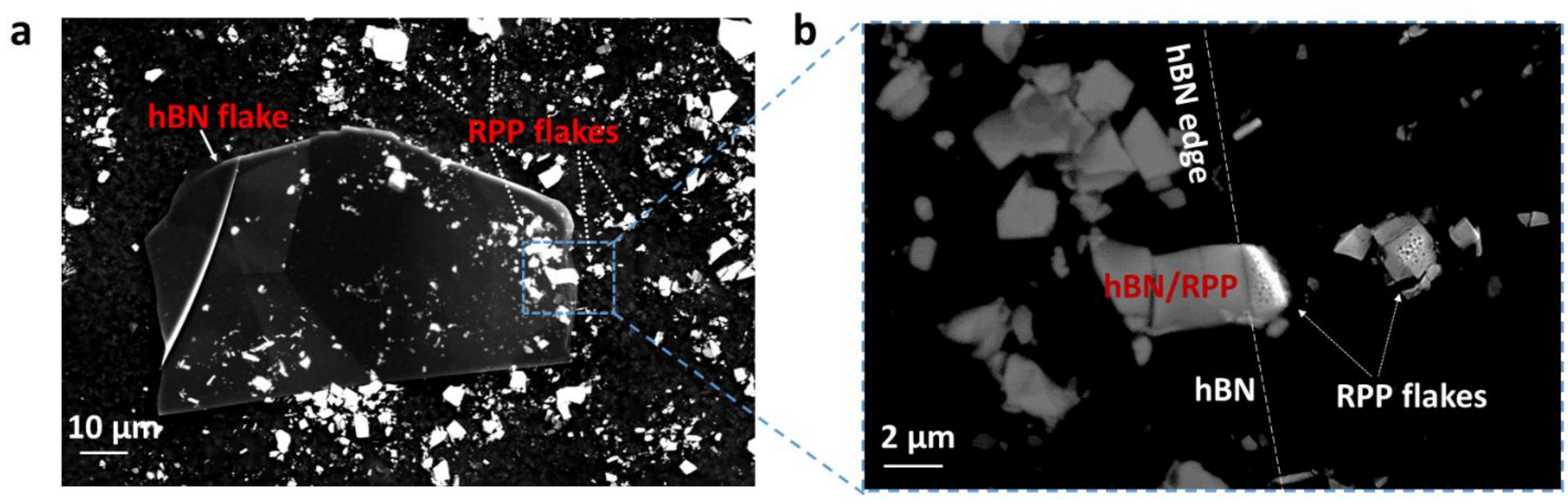

Supplementary Figure 6: Protection efficiency of hBN encapsulation of RPP flakes against an intense electron-beam condition, at a high current of 16 nA, acceleration voltage of 10 kV and acquisition time of 500 ms in the SEM apparatus. (a) Secondary-electron SEM image of a large hBN flake exfoliated on multiple RPP flakes. (b)The magnified view of the partially covered RPP flake at the edge of hBN flake. Uncovered parts of RPP flakes (right side) are drastically degraded, while encapsulation by hBN flake has significantly hindered the electron-beam-induced degradation.

To investigate the efficiency of exciton excitation by electron-beam in unencapsulated RPP flakes, while keeping a low degradation rate, we change the acceleration voltage (10 kV, 15 kV and 20 kV in Supplementary Figure 7) at a fixed low current of 200 pA in the mirror-collected CL spectroscopy. Efficient excitation of bulk excitons along the scanning length by the electron-beam (shown by blue arrows) is confirmed for acceleration voltage of 20 kV (Supplementary Figure 7a), as opposed to lower acceleration voltages that lead to excitation of excitons merely at the flake edge (Supplementary Figure 7b and 7d). Moreover, Supplementary Figure 7 does not show any detectable degradation trace either in the secondary electron images of RPP flakes, nor in the CL peak intensity when using low electron currents. Hence, we fix the acceleration voltage at 20 kV and the electron-beam current below 200 pA, where the current is measured at the sample position, to investigate RPPs and the hBN/RPP heterostructures by cathodoluminescence.

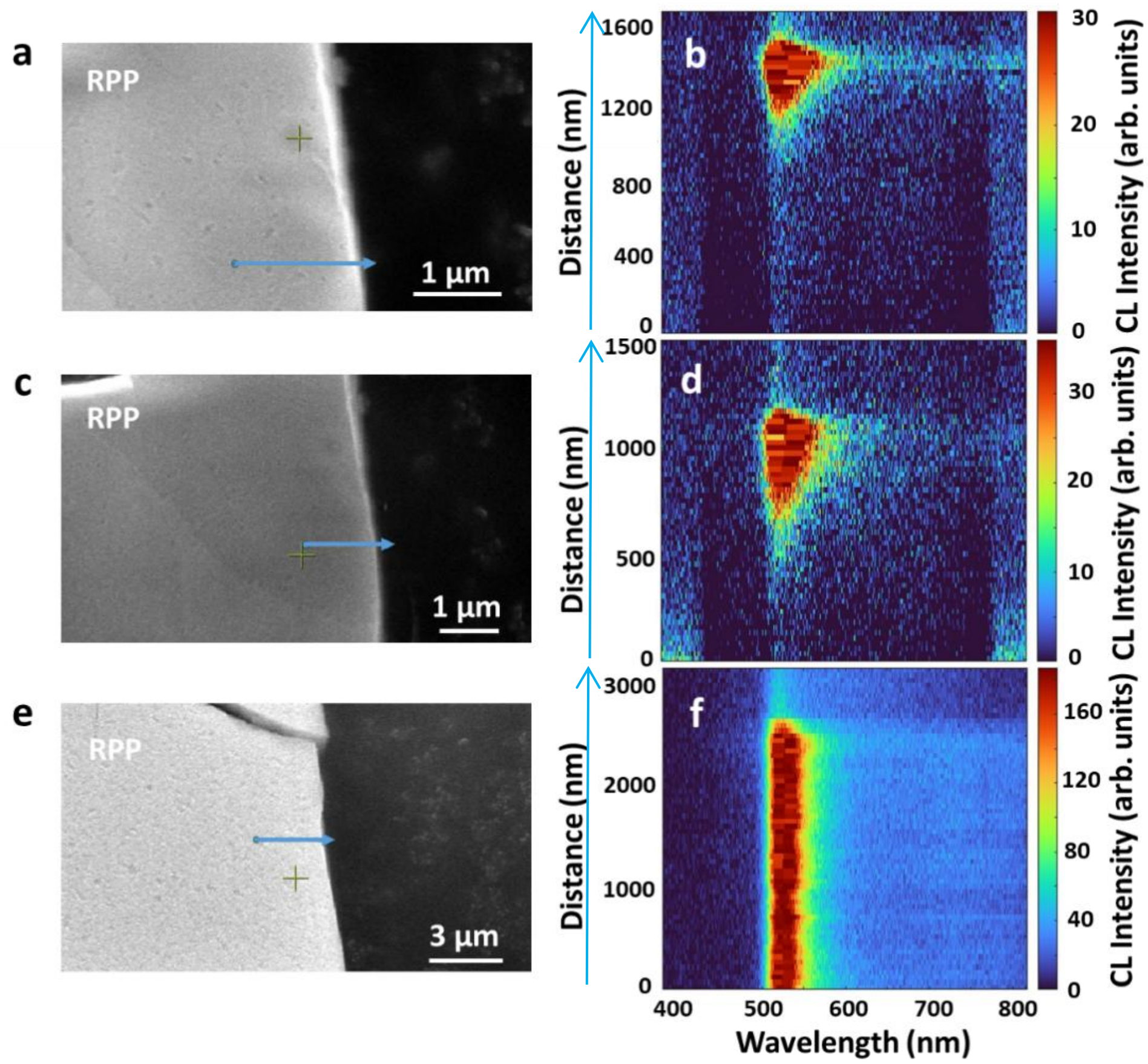

Supplementary Figure 7: Exciton excitation efficiency of the unencapsulated RPP flakes at different acceleration voltages explored by the mirror-collected CL spectroscopy. Scanning the electron-beam along the blue arrows shown in the left images in parts (a, c, e). Current level is fixed at 200 pA for all measurements to avoid the electron-beam-induced degradation in RPP. The measured CL spectra along the scanning length at acceleration voltages of (b) 10 kV, (d) 15 kV, and (f) 20 kV. Acceleration voltages below 20 kV leads to exciton excitation just at flake edges, while (f) proves efficient excitation of bulk excitons in RPP flakes without considerable degradation.

Supplementary Note 6: Tight-binding model for defect–defect interactions and photon-mediated defect networks

To rationalize the long-range energy redistribution observed experimentally, we model the ensemble of optically active defects in hBN as a network of radiatively coupled two-level systems (TLSs). Excitation transfer between defects is described within a tight-binding framework, where coupling between emitters is represented by an effective hopping amplitude.

1. One-dimensional toy model

We first introduce a minimal one-dimensional chain of N TLSs described by the Hamiltonian

$$\hat{H} = \hbar\omega_0 \sum_{n=1}^{N} \hat{a}_n^\dagger \hat{a}_n + \hbar J \sum_{n=1}^{N} \left(\hat{a}_n^\dagger \hat{a}_{n+1} + \hat{a}_n \hat{a}_{n+1}^\dagger\right) , \qquad (1)$$

Where $\hbar$ is the reduced Planck's constant, $\hbar\omega_0$ and $\hbar J$ are the energies of the TLSs at the excited state and the hopping amplitude between the neighboring emitters, respectively. The operators $\hat{a}_n$ and $\hat{a}_n^\dagger$ are the annihilation and creation operators associated with the nth TLS.

Restricting the dynamics to the single-excitation manifold, the wavefunction of the system is written as a coherent superposition of localized excitations, expressed as $|\Psi\rangle = \sum_{n=1}^{N} c_n(t)|\varphi_n\rangle$. Inserting this ansatz together with Equation (1) into the time-dependent Schrödinger equation yields the coupled equations of motion,

$$i\dot{c}_n = \omega_0 c_n + J c_{n-1} + J c_{n+1} , \qquad (2)$$

with appropriate boundary conditions at the ends of the chain.

The system of Equation (2) is solved numerically using a fourth-order Runge-Kutta method. For $\omega_0 = 2\pi, J = 0.5$, and $N = 500$, and the initial conditions $c_1(0) = 1, c_{n\neq 1}(0) = 0$, the calculated time-dependent amplitudes are shown in Supplementary Figure 8. As seen, the amplitude at site $n = 1$ is rapidly depleted, while the excitation is transferred, with a finite delay, to the other elements of the array. The amplitudes exhibit pronounced oscillations in time, originating from constructive and destructive interference between excitation pathways involving neighboring sites.

Since the model describes a closed system, the excitation is eventually reflected from the ends of the finite chain, leading to revivals at long times. In the following, we focus on the short-time regime, before such boundary reflections occur, which is the relevant regime for comparison with experiment.

Supplementary Figure 9 shows the maximum amplitudes extracted for each site n, obtained from the diagonal cut of the time-site map in Supplementary Figure 8. The site-dependent envelope follows closely an exponential decay $a_0 \exp(-t/\tau) + b_0$, with the fitting parameters equal to $a_0 = 0.41$, $\tau = 51.78 \cdot 2\pi\omega_0^{-1}$, and $b_0 = 0.2$. This exponential spatial decay arises from multipath interference associated with forward and backward hopping processes in the chain, which progressively funnel the excitation across neighboring elements. The underlying interference

patterns are directly visible in the time-dependent amplitudes (see inset of Supplementary Figure 9).

This behavior is closely analogous to free-induction decay, where the superposition of multiple coherent pathways with different phases leads to an apparent exponential attenuation of the observable amplitude, even in the absence of intrinsic dissipation. In the present tight-binding network, the decay emerges from the coherent redistribution of excitation probability over an increasing number of sites.

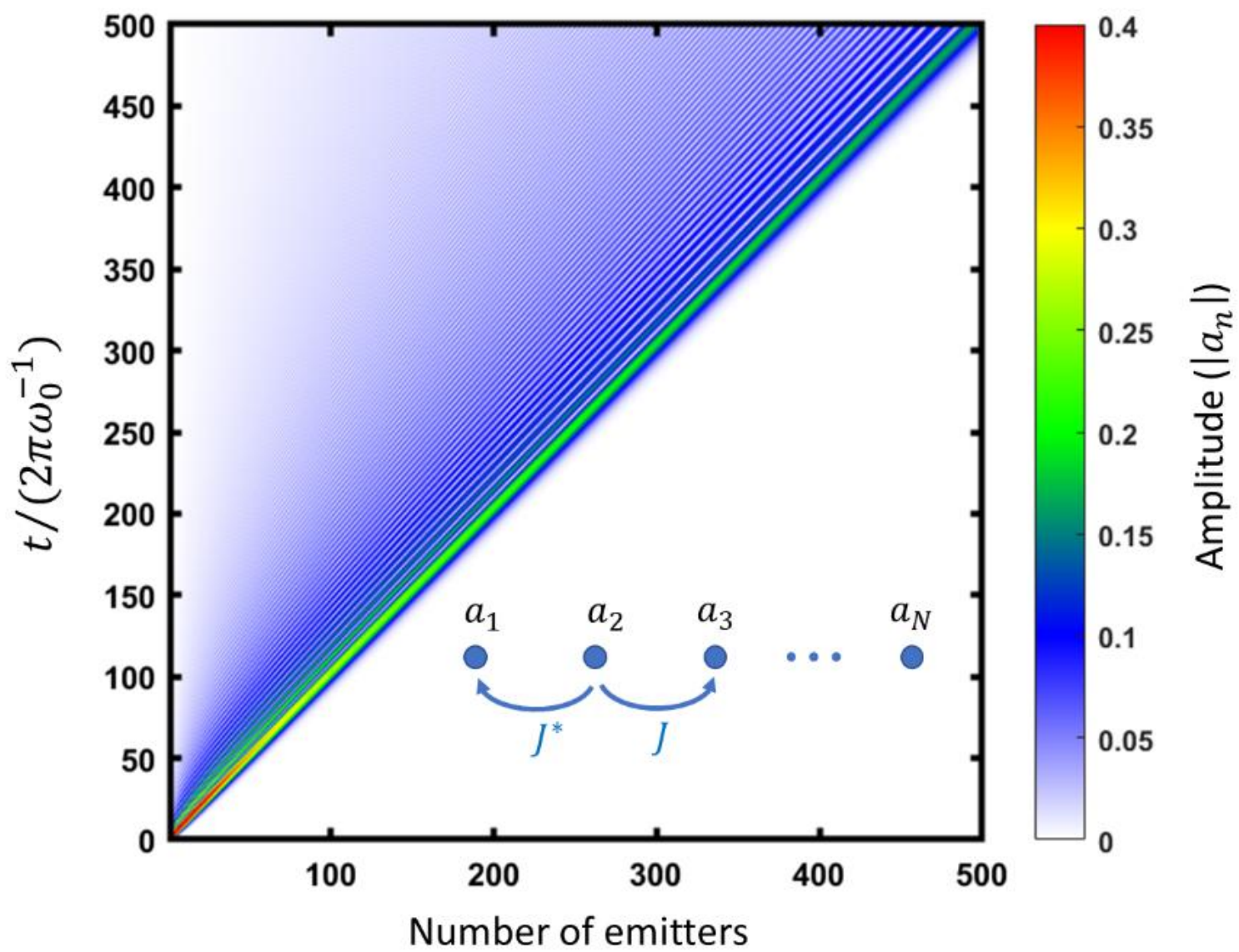

Supplementary Figure 8. Calculated amplitudes $a_n(t)$ as a function of time. For the introduced parameters see the text.

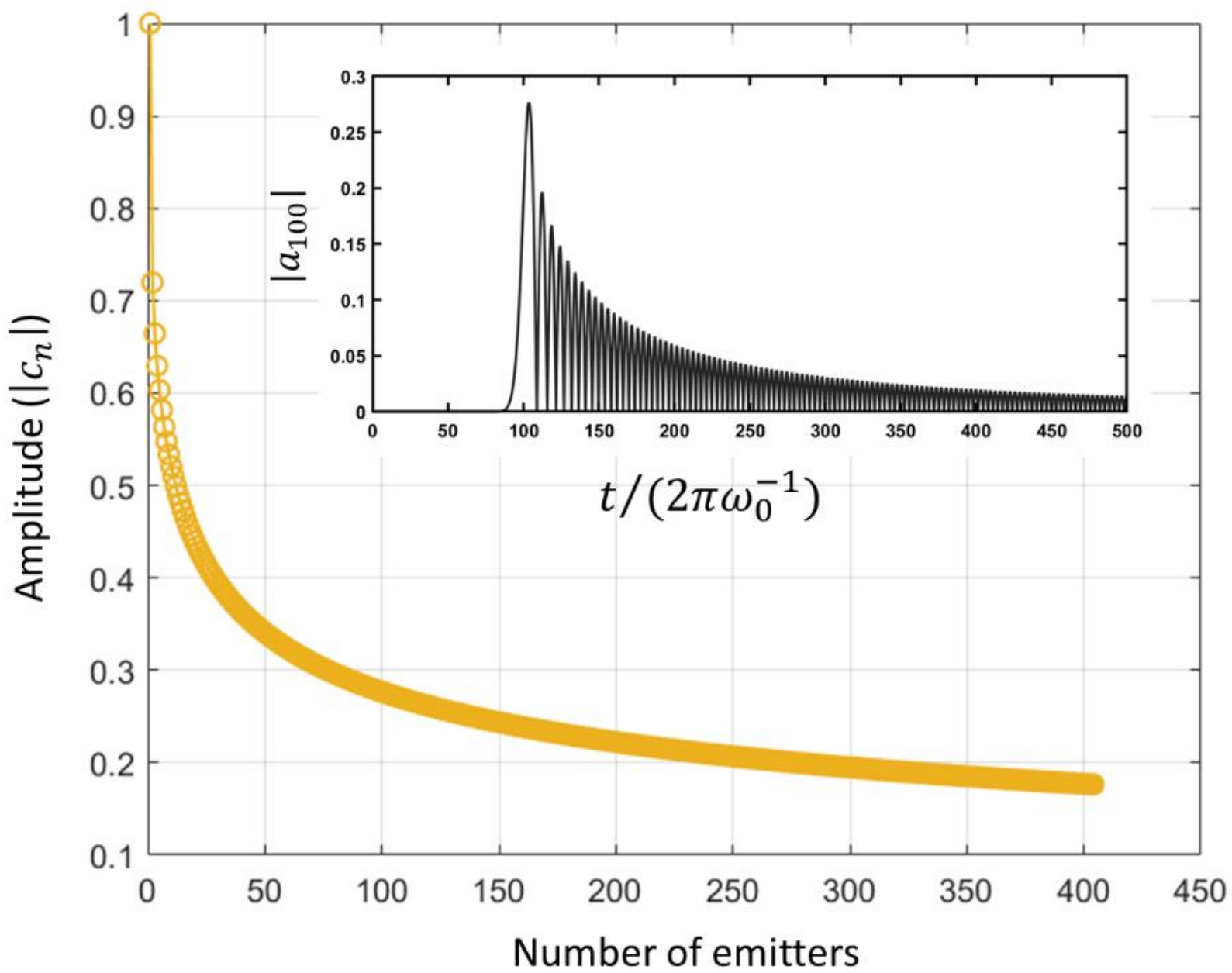

Supplementary Figure 9. Peak site amplitude versus location. The inset demonstrates the time-dependent amplitude at cite $n = 100$, which exhibits oscillations due to the multipath interference between the energy funneled from neighboring elements to this site.

2. Extension to a two-dimensional disordered defect network

Real hBN flakes contain defects distributed in two dimensions with positional disorder. In such a network, the number of available excitation pathways and interference channels is significantly larger than in the 1D toy model, and an effective exponential spatial decay is therefore expected to be even more pronounced.

To capture this situation, we consider a 2D ensemble of emitters randomly distributed according to a Gaussian distribution with average inter-defect spacing $\bar{s}$ and variance $\Delta s = 0.1\bar{s}$. In contrast to the nearest-neighbor coupling assumed above, we now include **long-range dipole–dipole interactions with retardation**, allowing for near-field, intermediate-field (Fraunhofer), and far-field radiative coupling.

The electric field produced by a dipole emitter contains terms that scale as r^{-1}, r^{-2}, r^{-3}, corresponding to far-field, intermediate-field, and near-field radiative contributions, respectively. The electric field emitted by dipole is given by $E_\Theta = \frac{d}{4\pi}\left(\frac{i\omega_0\mu_0}{r} + \frac{\eta\omega_0}{r^2} + \frac{1}{i\varepsilon_r\varepsilon_0 r^3}\right)e^{ikr}$ [5]. The resulting interaction energy between two parallel dipoles separated by distance r_{mn} can therefore be written schematically as:

$$\hbar J_{mn} = \frac{d^2}{4\pi\varepsilon_0\varepsilon_r}\frac{\cos(kr_{mn})}{r_{nm}^3} + \frac{\omega_0\eta d^2}{4\pi}\frac{\cos(kr_{mn} + \pi/2)}{r_{mn}^2} + \frac{\omega_0^2\mu_0 d^2}{4\pi}\frac{\cos(kr_{mn})}{r_{mn}}. \quad (3)$$

Using this distance-dependent coupling, Equation (2) is generalized to a fully connected network:

$$i\dot{a}_m = \omega_0 a_m + \sum_{n \neq m} J_{mn} a_n \ , \qquad (4)$$

The hopping amplitude in the first term of Equation (3) is consistent with the **Förster-type resonant energy transfer mechanism where the** emitters interact via their **transition dipoles**. The second and third terms are the generalization of the dipole-dipole interactions when retardation is considered as well, such as in the case of the photon-recycling effect. In Equation (3) d is the transition dipole that can be calculated as $\sqrt{\frac{3\pi\varepsilon_0\hbar c^3}{n\omega_0^3\tau}}$, ε_0 is the free-space permittivity, $\hbar$ is the reduced Planck's constant, $n = 2.1$ is the refractive index of hBN, $k = nk_0$ is the wavenumber in hBN, and τ is the decay time taken as approximately 40 ns (Figure 1e of the main text).

The system is numerically solved using the Runge-Kutta method. The initial condition is $a_1 = 1$ and $a_{n\neq 1} = 0$, where the $n = 1$ point is positioned in the center of the network. We numerically solve this system for networks containing up to $4 \cdot 10^5$ emitters with average spacing of 80 nm − 150 nm, consistent with experimentally estimated defect densities. The excitation is initially localized near the center of the network (Supplementary Figure 10a). The transition frequency is set to $\omega_0 = 2\pi c\lambda_0^{-1}$, where $\lambda_0 = 530\ nm$ is the emitter wavelength.

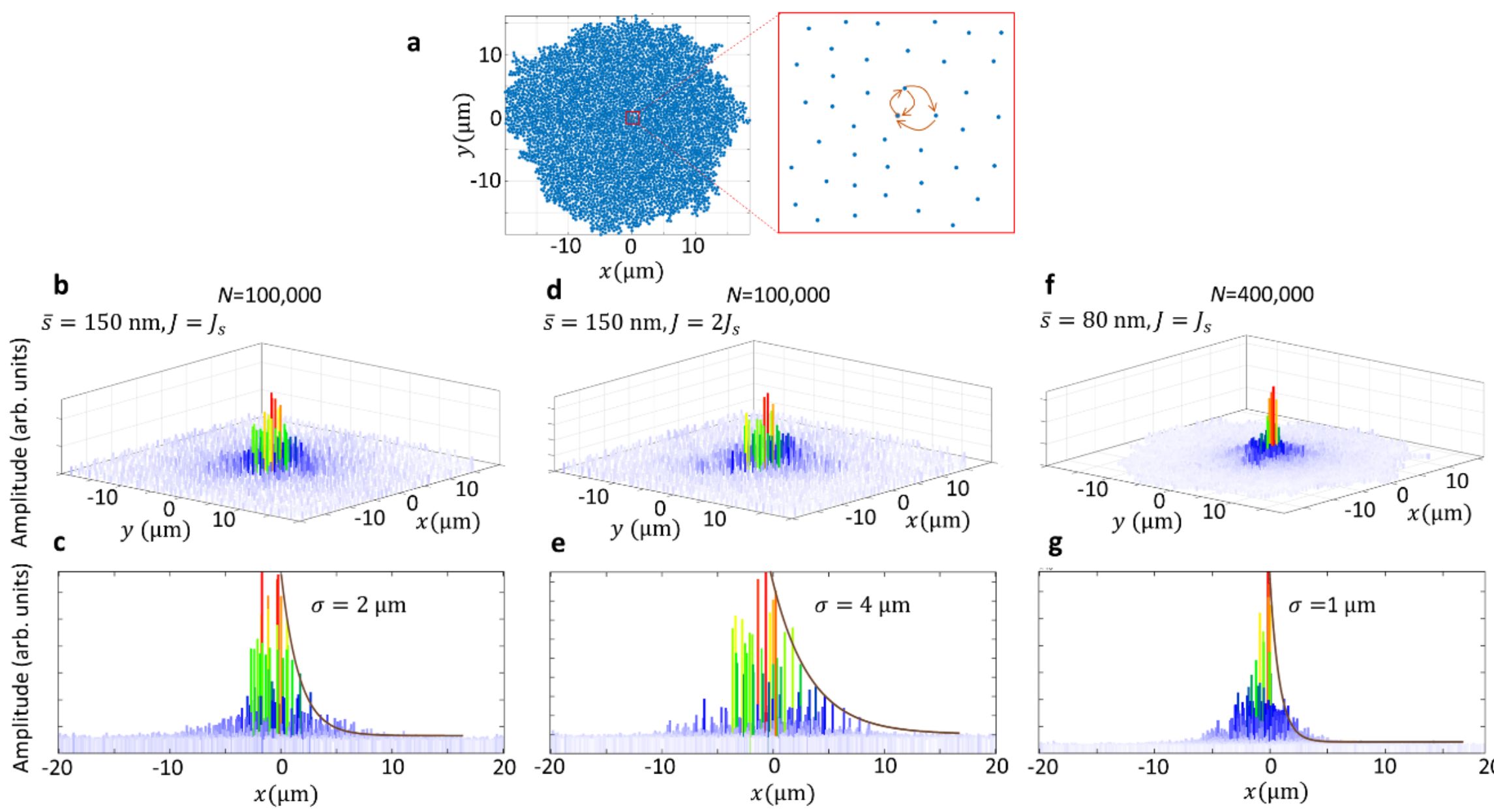

Supplementary Figure 10. (a) A 2D network of emitters where the emitters are positioned randomly, following a Gaussian distribution with an average spacing of 150 nm. In all panels, only a selected set of scattered points are demonstrated. (b-g) The averaged amplitudes distributed over the network for different depicted emitter numbers, average distances between the points, and hopping amplitudes. Panels b, d, and f show the 3D configurations, whereas c, e, and f demonstrate the amplitudes along the $y = 0$ axis, demonstrating an exponential decay.

To allow for a practical simulation size and at the same time to avoid reflection from the ending of the array, we consider a hopping amplitude at the maximum value of $J_{s,nm} = 0.01J_{nm}$. The results are demonstrated in Supplementary Figure 10b and c, where we observe an exponential decay of $\sigma = 2$ μm in the amplitude. Next, we increase the hopping amplitude to $J = 2J_s$ (Supplementary Figure 10d and e), where the decay length is doubled as well. Moreover, by decreasing the average distance between emitters from 150 nm to 80 nm, while keeping the hopping amplitude the same, the decay length is decreased to only 1 μm.

In all cases, the envelope of the excitation probability exhibits an approximately exponential decay with distance from the excitation point. Key trends emerge:

- Increasing the effective hopping amplitude increases the decay length.
- Decreasing the average inter-defect spacing (i.e., increasing defect density) can **reduce** the decay length due to stronger interference and redistribution over many competing path-ways.

This counterintuitive behavior matches our experimental observation that Ar-treated samples with higher defect densities exhibit **shorter** transport lengths. The reason for this is elaborated below.

Because realistic samples contain orders of magnitude more emitters (estimated 10^8), full-scale simulations are computationally infeasible. The reduced-size simulations therefore serve as a **scal-ing model**, demonstrating the physical mechanism and trends rather than reproducing absolute numbers. Extrapolation to experimentally relevant densities yields decay lengths on the order of 200 μm, consistent with measured values.

3. Timescales and room-temperature operation

The characteristic timescale for excitation redistribution in the simulations is on the order of pico-seconds, which is much shorter than the measured defect radiative lifetime (nanoseconds). Energy can therefore be redistributed across the network before spontaneous decay occurs. Since the dom-inant coupling channels in this regime are radiative and retarded dipole interactions, rather than fragile wavefunction overlap, the mechanism is not suppressed at room temperature.

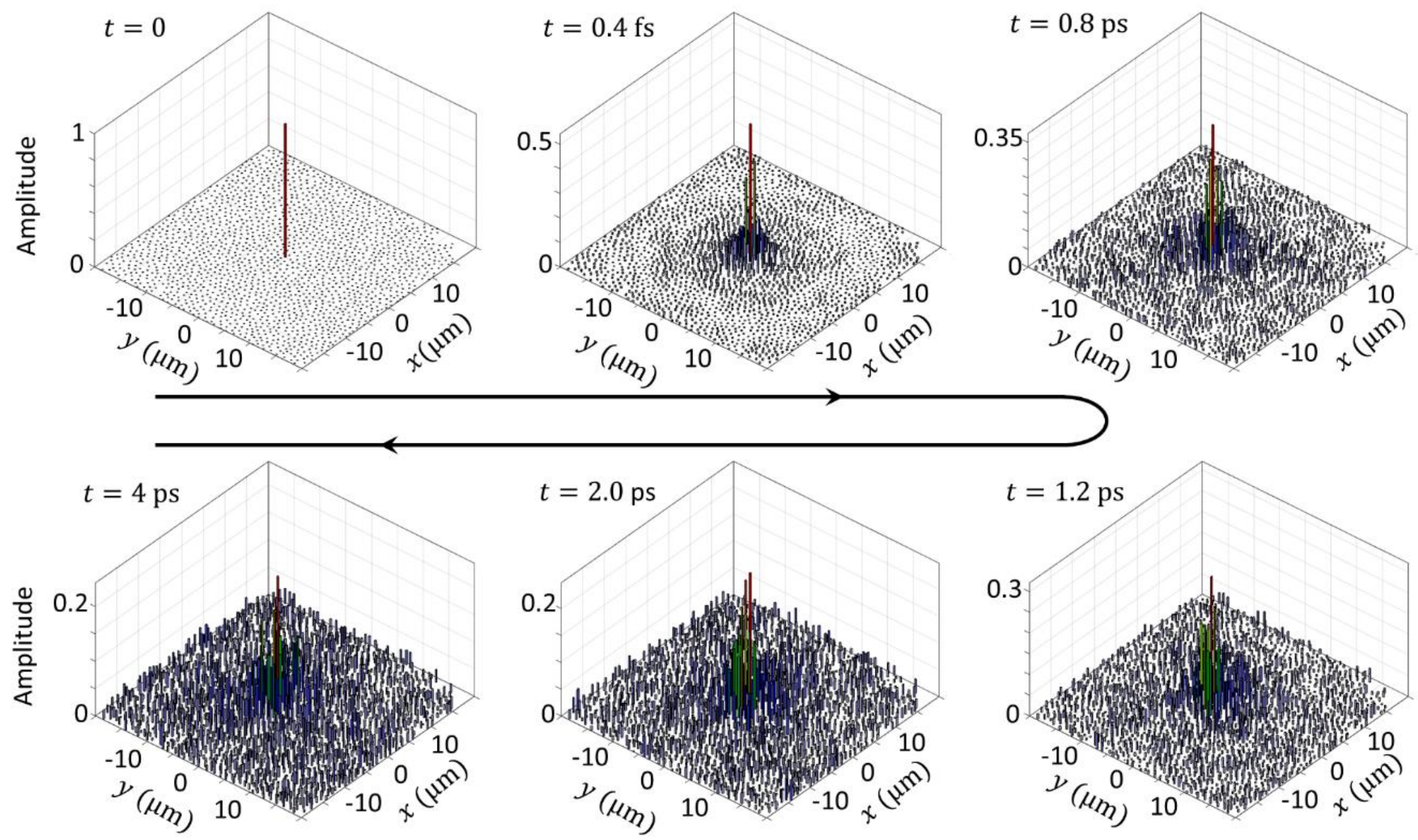

Supplementary Figure 11. Snapshots of the amplitude redistribution over the network at depicted times.

4. Experimental consistency: defect-density dependence

To experimentally test the predicted density dependence, we increased the defect concentration in hBN using Ar plasma treatment. Cathodoluminescence measurements show that samples with higher defect densities exhibit significantly shorter energy-transfer decay lengths (Supplementary Figures 12 and 13), in agreement with the model prediction that stronger coupling and increased pathway interference can reduce the effective transport length.

The hBN samples were prepared using mechanical exfoliation and subsequently subjected to argon plasma treatment using a Henniker Plasma HPT-100 system equipped with a low-pressure radio-frequency (RF) plasma generator operating at power of 20 W and frequency of 40 kHz. Prior to Ar plasma exposure, the chamber was evacuated to a base pressure of 18 Pa. The hBN sample was exposed to the Ar plasma at pressure of 23 Pa, for various time durations, and subsequently explored using CL spectroscopy, to find the best configuration and duration time.

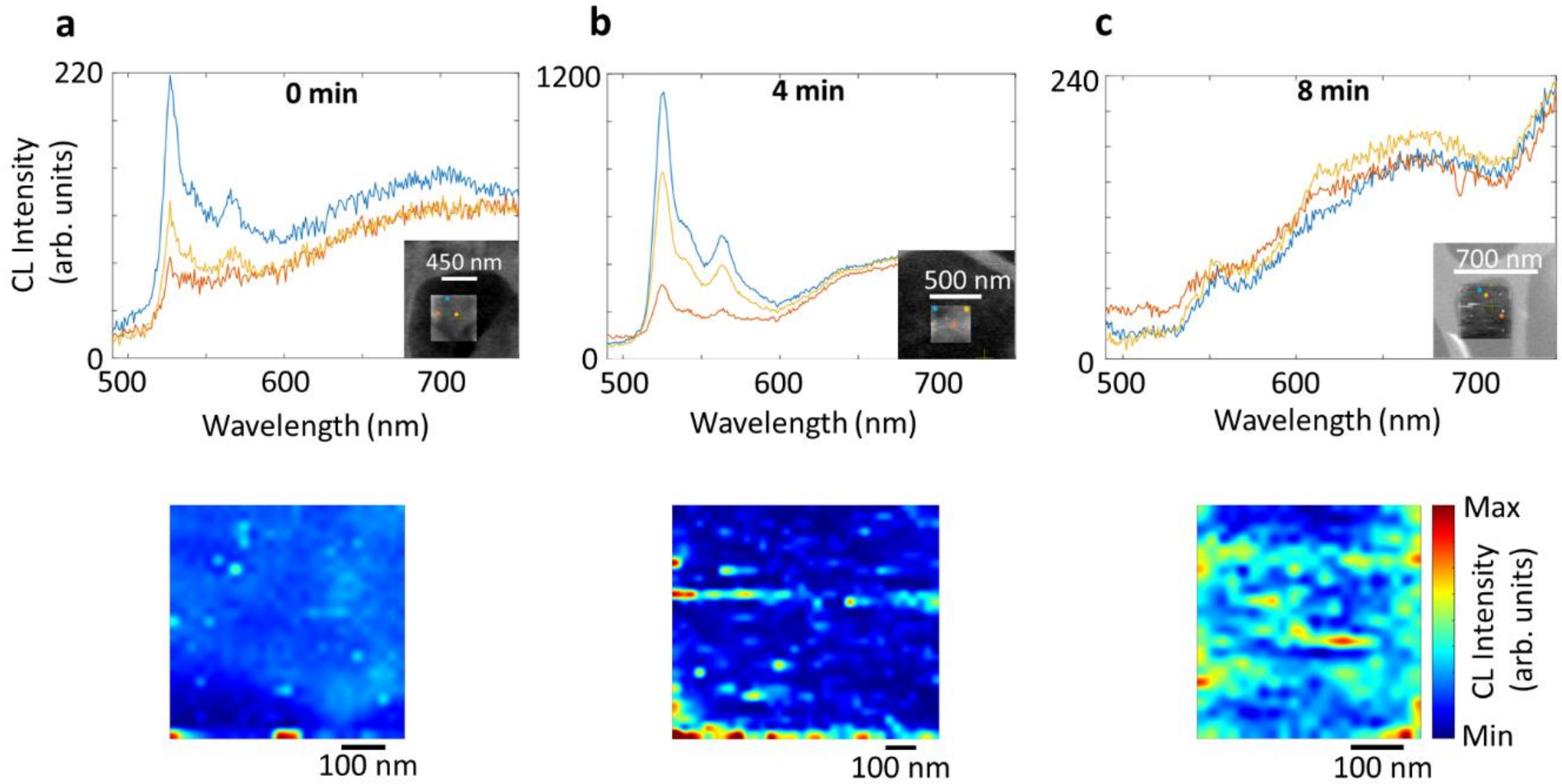

Supplementary Figure 12. Cathodoluminescence spectra (top row) and hyperspectral images at 537 nm emission (bottom row). The insets show the SEM images and the marked positions where the CL spectra are taken from. (a) An untreated hBN flake, Ar-treated hBN flakes with (b) 4 min, and (c) 8 min treatment durations.

The sample with 4 min treatment duration was prepared and transferred on top of RPP flakes. The heterostructure is explored with our fiber-based CL setup, demonstrating a decay length of around 58 μm, significantly shorter than the decay length in the hBN/RPP heterostructures with pristine hBN flakes.
The observed reduction of the effective decay length with increasing hopping strength or defect density arises from the interplay of multipath quantum interference and the different distance-dependent contributions to the coupling terms in Equation (3) (Supplementary Figure 14). As the number of available pathways increases, the excitation probability is redistributed over many

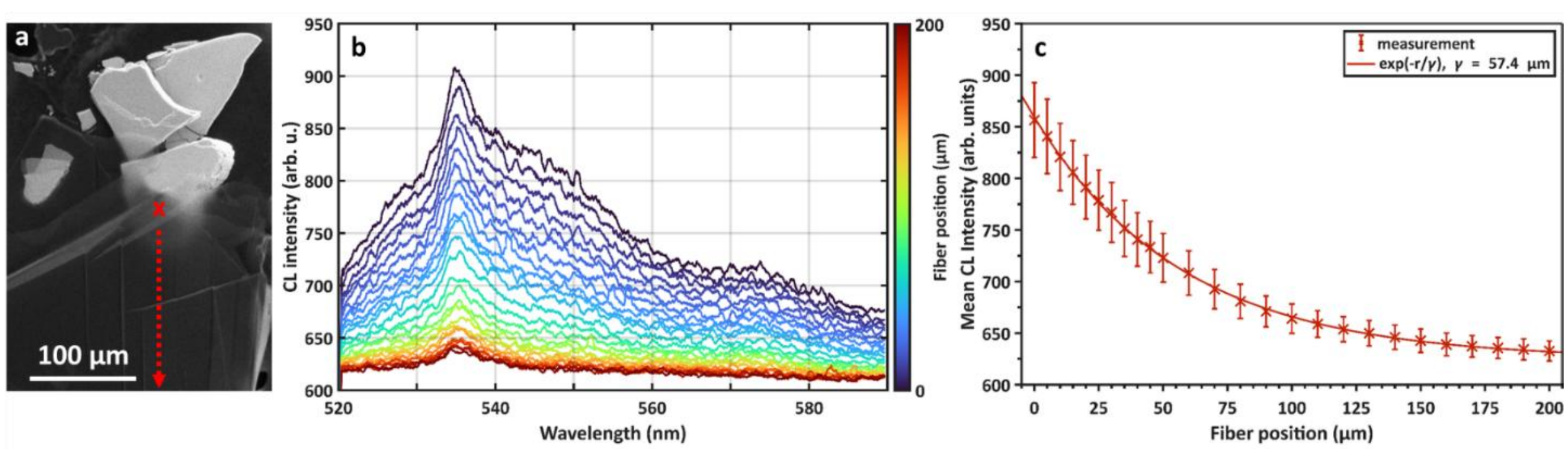

Supplementary Figure 13. (a) scanning electron microscope image of an Ar-treated hBN/RPP heterostructure positioned on carbon. The X marker represents the excitation position of the electron beam, while the red dashed-arrow indicates the scanning-path of the optical fiber. (b) Measured cathodoluminescence spectra along the path, where from dark blue to dark red, shows the path along the scanning position from top to down. (c) Peak cathodoluminescence intensity versus the fiber position, which manifests an exponential decay with the decay length of around 58 μm.

competing channels, which enhances interference effects and leads to a faster spatial attenuation of the envelope.

For very small inter-defect separations ($\lesssim 20$ nm), the quasi-static near-field term in Equation (3), which scales as r^{-3} and is independent of the frequency of the emitter, becomes dominant. In this regime, the interaction approaches the conventional non-radiative Förster-type limit. However, such ultrashort separations are not representative of the defect configurations investigated here. At these distances, individual defects would no longer act as isolated emitters but instead form clusters with distinct collective emission spectra, as observed experimentally for heavily Ar-treated samples (Supplementary Figure 12c).

The defect densities in the samples used for the long-range transport measurements correspond to larger average spacings, where the retarded intermediate-field and far-field radiative terms in Equation (3) provide significant or dominant contributions to the coupling. Therefore, the correlations responsible for the observed energy redistribution are best described by these photon-mediated interaction terms rather than by purely quasi-static dipole-dipole coupling.

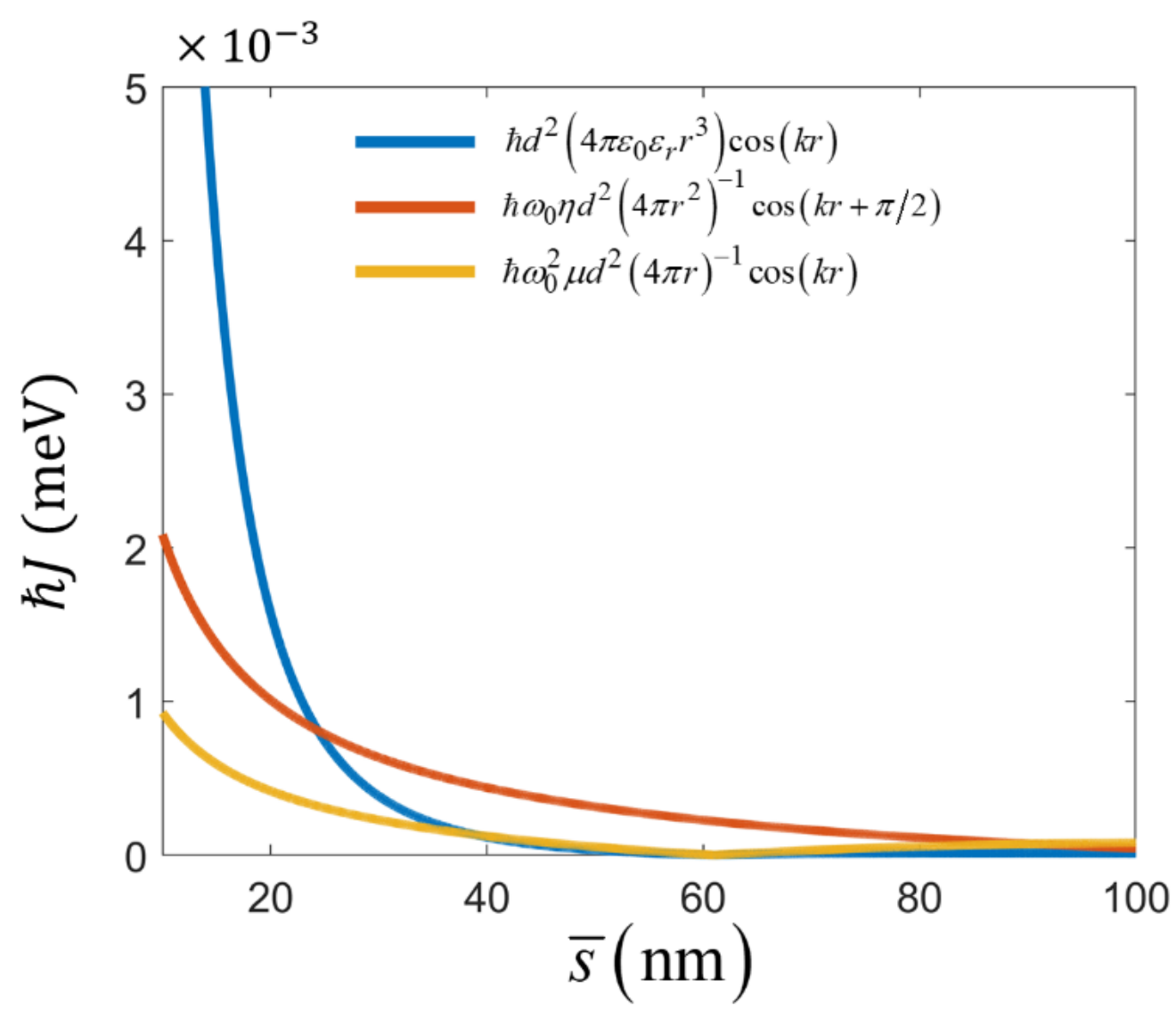

Supplementary Figure 14. Different constituents of the hopping amplitude versus the average distance between emitters.

Supplementary Note 7: Waveguiding behavior of hBN/RPP/carbon and hBN/carbon heterostructures

To interpret our experimental results, we calculated the energy–momentum map of an hBN/RPP/carbon heterostructure and compared it with a region without the RPP flake (schematic illustrated in Supplementary Figure 15c), as shown in Supplementary Figure 15a. The hBN/RPP/carbon sample exhibits a waveguiding mode captured in the RPP layer extending to large $k_{\parallel}$ outside the free-space light cone, which is modified in the vicinity of the RPP exciton energy, due to strong coupling, whereas the hBN/carbon sample lacks the guided mode response. This

result demonstrates that RPPs can sustain optical modes within the film that hybridize with excitons, and giving rise to guided exciton-polaritons around the excitonic energy range[6].

Supplementary Figure 15b demonstrates simulated maps of the magnetic-field component H_y evaluated at the three marked conditions I–III in energy-momentum map at fixed energy of 2 eV **(**Supplementary Figure 15a**).** However, since both RPP and carbon have a higher refractive index than hBN in the relevant energy range, the lateral propagation is supported by the higher-index film section**,** while the field decays evanescently into the surrounding lower-index region and into the superstrate (energy dependent complex permittivity of carbon and hBN demonstrated in Supplementary Figure 15d). As the excitation condition moves along the dispersive branch from I to III (larger $k_{\parallel}$), the mode becomes more guided in RPP layer, with enhanced transverse confinement and a longer-range propagation.

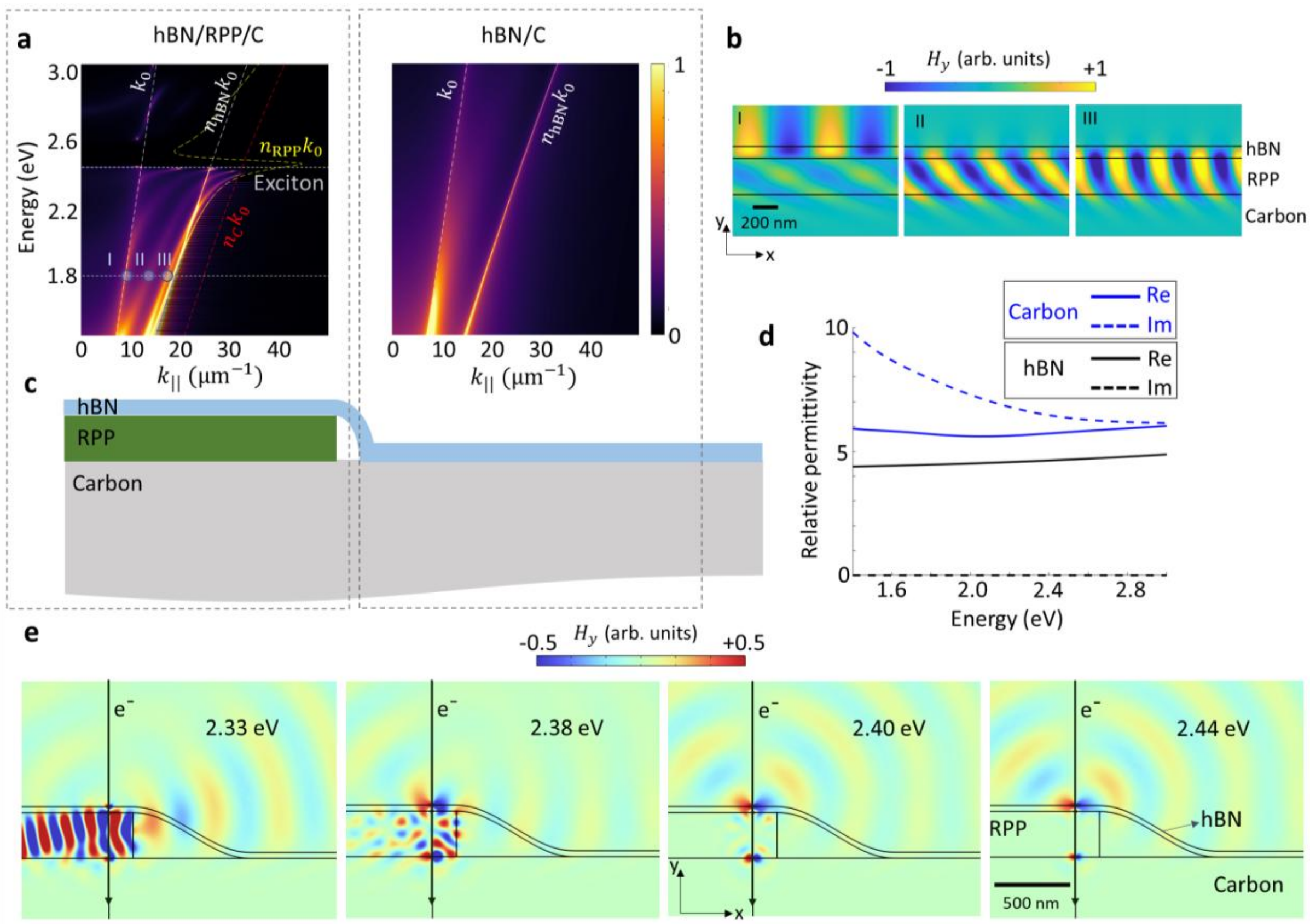

Supplementary Figure 15. **Calculating the optical responses of the hBN/RPP/C heterostructure.** (a) Energy-momentum maps comparing the hBN/RPP/carbon heterostructure (left) with a hBN/carbon stack (right). Dashed lines indicate the free-space light line and the light line in the constituent media. (b) Simulated H_y field maps evaluated at the three points I, II, III indicated in (a), demonstrating confined guided propagation in the RPP layer. (c) Schematic of the simulation domain, demonstrating partial coverage of hBN flake over RPP and carbon. (d) Real and imaginary parts of the relative permittivity used for carbon and hBN. (e) Snapshots of the indued magnetic field at selected energies near the excitonic energy of the RPP after electron irradiation, showing absorption at the excitonic energy (2.44 eV) and energy-dependent propagation of the optical modes at the heterostructure at lower energies.

Next, we simulate the optical response of the structure per electron excitation, using the RF Module of COMSOL, solving Maxwell's equations in real space and in the frequency domain. The swift electron was modeled as an oscillating current along a straight line representing the electron trajectory, where the electron velocity is considered to be $v_e \approx 0.27c$. The permittivities of sub systems were taken as experimentally reported values as following: Carbon and hBN were taken from references [7,8] (see Supplementary Figure 15d), and the RPP permittivity was taken from the spectroscopic ellipsometry results reported elsewhere [9].

The maps in Supplementary Figure 15e show cross-sections of the induced magnetic field H_y at energies $E = 2.33$ eV, 2.38 eV, 2.40 eV, and 2.44 eV (exciton energy), or $\lambda = 532$ nm, 521 nm, 517nm, and 508 nm correspondingly, for an electron traversing the heterostructure. At energies below the exciton resonance (2.33 eV to 2.40 eV), the electron launches propagating guided modes in the RPP region, and produces oscillatory, and decaying responses and longer propagation. However, close to the exciton energy, where absorption peaks (here around 2.44 eV), the guided response is attenuated and the propagation length shortens, with increased attenuation (enhanced dissipation) of the waves inside RPP near the excitonic resonance. Therefor here at the excitonic energy this pronounced absorption in the RPP layer and reflection from the outer boundary are observed.

We further calculated E-$k_\parallel$ maps of free-standing hBN slabs with thicknesses $d = 50$ nm, 100 nm, and 150 nm to quantify the effect of hBN thickness in exciting waveguiding modes. As demonstrated in Supplementary Figure 16. A free-standing dielectric slab acts as a **planar waveguide** for optical modes which propagate in-plane with the propagation constant of $k_\parallel$. As the slab thickness increases, the number of guided waves and their dispersion alter. Furthermore, the field is confined in the transverse direction by the total internal reflection at the two hBN/air interfaces. This confinement requires solutions with $k_0 < k_\parallel < n_{\mathrm{hBN}} k_0$ (guided), where the field inside the slab is oscillatory but decays evanescently into the surrounding air. The corresponding spatial profile of the y-component of the magnetic field at selected energies have been ploted below each dispersion diagram as well, showing the enhanced confinement in the slab by increasing the thickness.

The guided waves inside the hBN flakes can be also experimentally detected using cathodoluminescence spectroscopy, where the transition radiation interferes constructively with the guided waves scattered from the edges of the flakes, and lead to interference fringes inside the energy-momentum map (Supplementary Figure 17). Similar behaviors have been used elsewhere as well to retrieve the propagation constant of waveguiding exciton polaritons in WSe_2 flakes [10].

Such interference fringes fully lack from the momentum-resolved maps shown in Figure 5 of the main text. In addition, we provide here hyperspectral and CL angle-resolved maps to better demonstrate the lack of coherent waveguiding modes and the interference effects that could result from them in our system (Supplementary Figure 18).

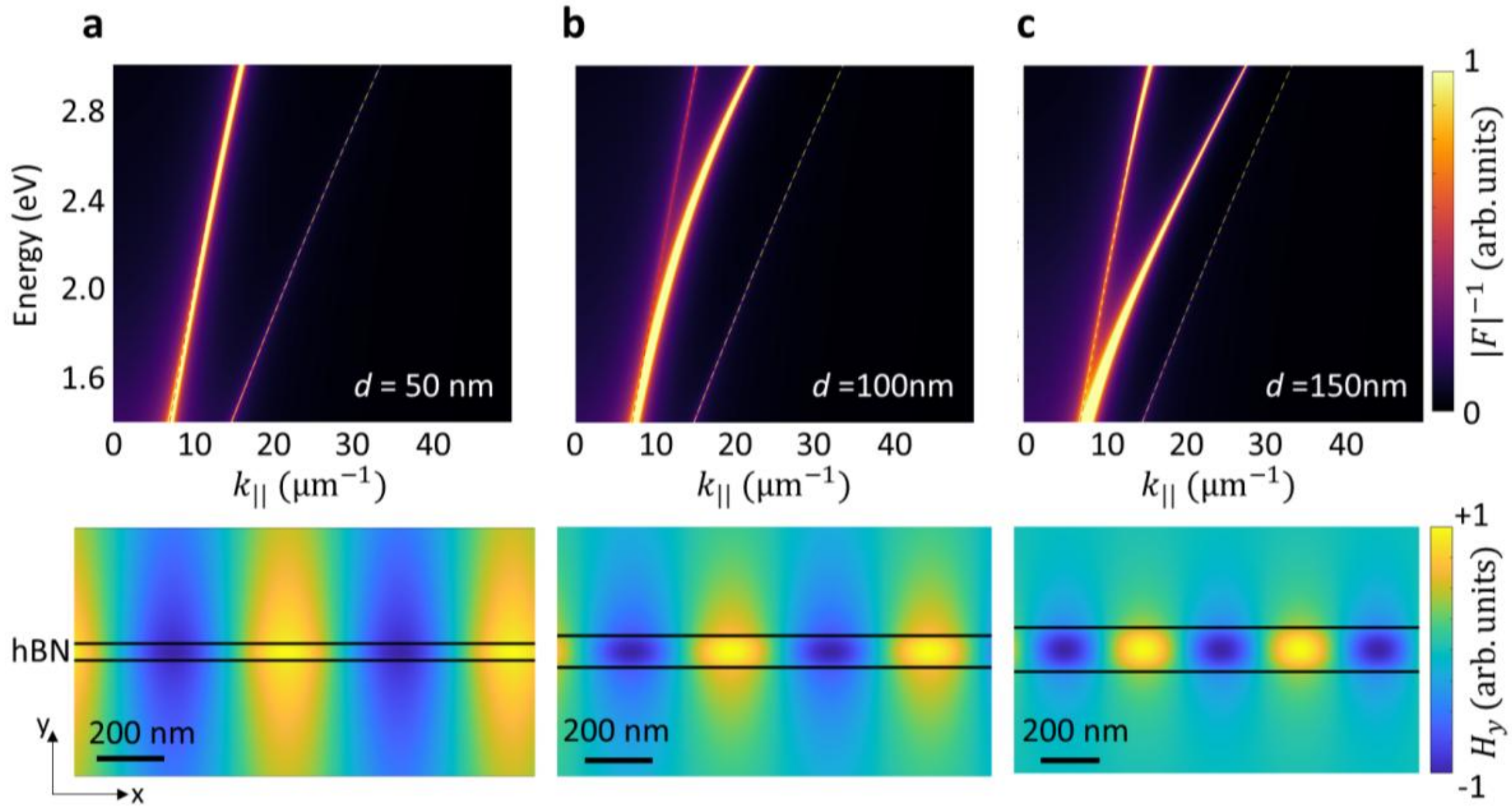

Supplementary Figure 16. **Slab modes in a free-standing hBN film.** (Top) Calculated energy-momentum maps of the optical response as a function of in-plane momentum $k_\parallel$ for free-standing hBN slabs with thickness $d = 50$ nm (a), 100 nm (b), and 150 nm (c). Dashed lines mark the vacuum light line (k_0) and the hBN light line ($n_{\mathrm{hBN}}k_0$); guided slab modes occur in the region $k_0 < k_\parallel < n_{\mathrm{hBN}}k_0$. (Bottom) Maps of the induced magnetic field H_y for each thickness, showing in-plane propagation with increasing transverse confinement and a more pronounced standing-wave character inside the slab as the thickness d increases.

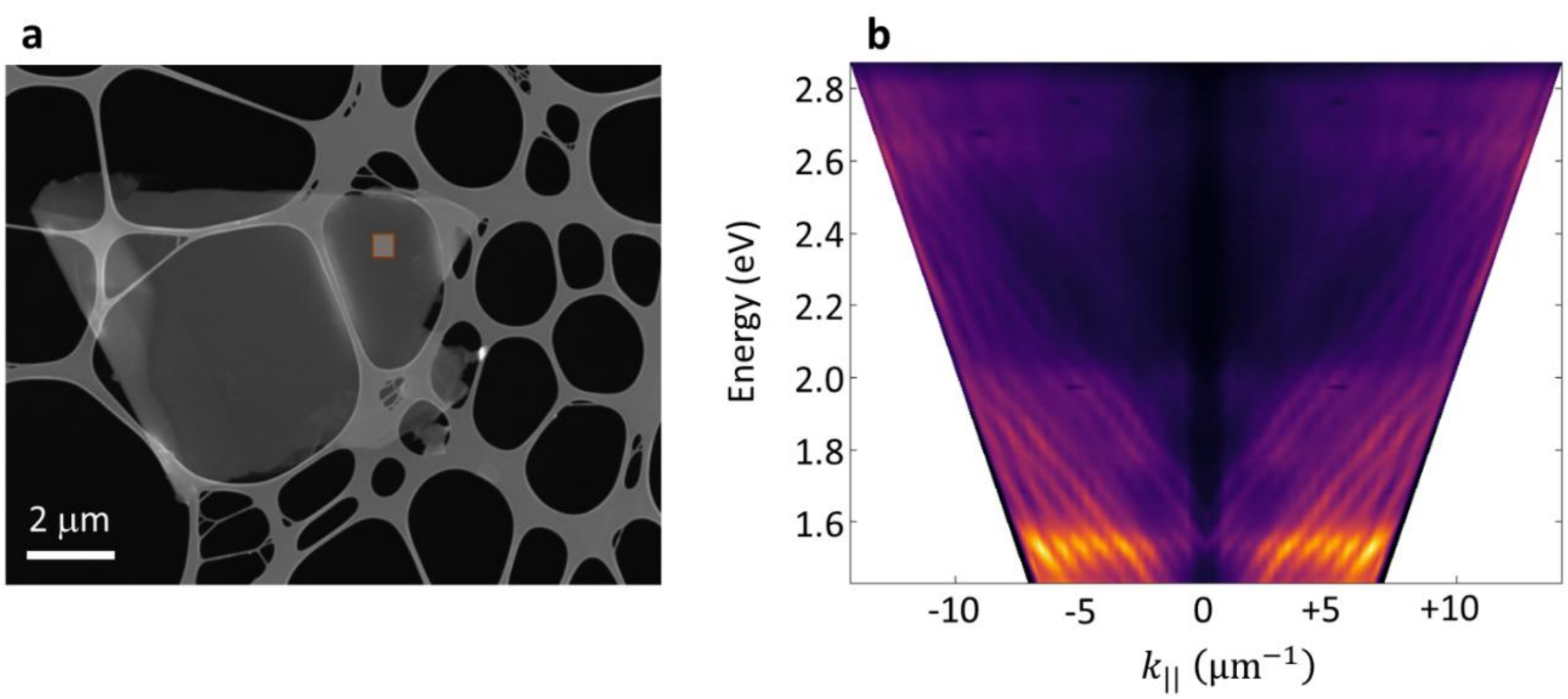

Supplementary Figure 17. (a) The SEM image of an hBN flake positioned on a holey carbon substrate, and (b) the energy-momentum map of the hBN flake, acquired at the position marked by the colored box in (a).

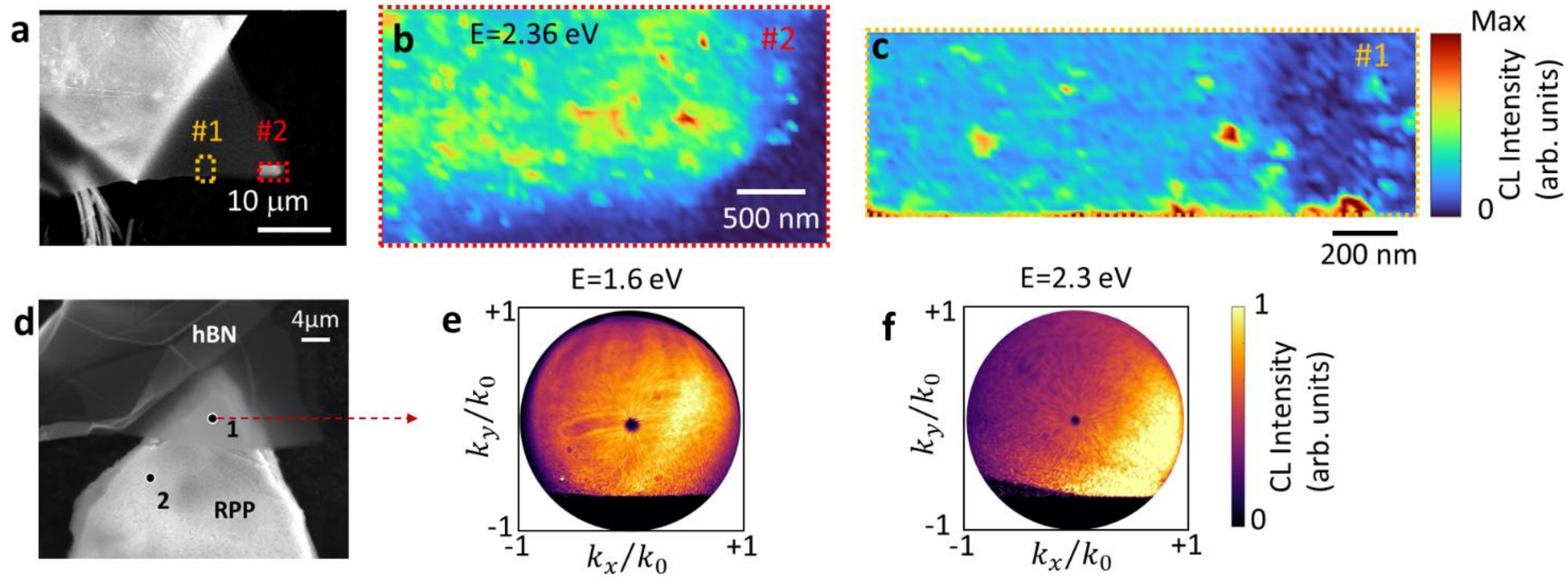

Supplementary Figure 18. (a) The SEM image of the hBN/RPP/C heterostructure. Hyperspectral images at $E = 2.36$ eV ($\lambda = 525$ nm), from the area marked by (b) dashed red box, and (c) dashed yellow box in panel (a). (d) The SEM image of an hBN/RPP/C heterostructure used for capturing the CL angle-resolved maps at depicted photon energies in (e) and (f).

Supplementary Note 8: Fitting procedure for the fiber-assisted CL spectroscopy measurements

In order to demonstrate that the measured CL spectra of the hBN/RPP heterostructure sustain a homogenous emission behavior and contain phonon sidebands, we have re-plotted and fitted the measurement results of Figure 1d for a better visuality. The re-plotted measurement results are shown in Supplementary Figure 19. Here, two different excitation spots were chosen to showcase the defect-mediated energy-transport mechanism, namely the CL spectra from the pristine RPP crystal (in green) and the stacked hBN/RPP heterostructure (in blue). The exact excitation spots by the electron beam are highlighted with circles by the same color in Figure 1b in the main text. The CL response of the pristine RPP crystals shows a rather broad emission peak around 530 nm which is attributed to the intrinsic excitonic mode. For further analysis, a Lorentzian function in the form of

$$f_{\text{Lorentzian}}(\lambda) = I_{\text{CL}} \cdot \left[1 + \left(\frac{\lambda - \lambda_{\text{c}}}{\gamma}\right)^2\right]^{-1} + C$$

was fitted to the data, where I_{CL} is the intensity amplitude of the measure CL signal, λ_{c} shows the central peak position, and 2γ represents the full width at half maximum (FWHM). The matching Lorentzian fit supports a homogenous emission profile of the investigated RPP crystal, where the fitted line profile reveals a peak position at 531 nm with a FWHM of 27.4 nm. For the stacked hBN/RPP heterostructure, the CL spectrum exhibits a much narrower and slightly red-shifted emission peak. Again, the Lorentzian function fits well with the measurement and this time reveals a peak position of 535.6 nm with a FWHM of roughly 4.9 nm. As we already explained in the main text, we attribute the red-shift to the coupling of the excitons inside the RPP crystal to the

defect center vastly spread across the overlaying hBN flake. Additionally, the CL spectra of the hBN/RPP heterostructure features two phonon sidebands due to presence of hBN defects around 550 nm and 570 nm, respectively, which are in relatively good agreement with the results obtained from mirror-assisted CL spectroscopy. To fully capture the measured CL spectrum of the heterostructure, we further fitted the Fermi-Dirac function

$$f_{\text{Fermi-Dirac}}(\lambda) = I_{CL} \cdot \left[\exp\left(\frac{\lambda - \lambda_0}{\kappa}\right) + 1\right]^{-1} + C$$

to each phonon sideband. Here, I_{CL} is the intensity amplitude of the measured CL signal, λ_0 is the position of the sideband, and κ represents the width of the sideband. Below, Table 1 shows the complete parameter set of the fitting procedure.

Table 1: Resulting parameters of the fitting procedure to compare the fiber-collected CL spectra from the pristine RPP and the hBN/RPP heterostructure, see Supplementary Figure 19. The used functions $f_{\text{Lorentzian}}(\lambda)$ and $f_{\text{Fermi-Dirac}}(\lambda)$ for fitting are mentioned in the text above.

Sample structure	Exciton peak position λ_c (nm)	Exciton peak width (FWHM) 2γ (nm)	Phonon sideband position λ_0 (nm)	Phonon sideband width κ (nm)
Pristine RPP flake (green)	530.8	27.4	---	---
Stacked hBN/RPP heterostructure (blue)	535.6	4.9	560.2<br>577.0	1.8<br>1.0

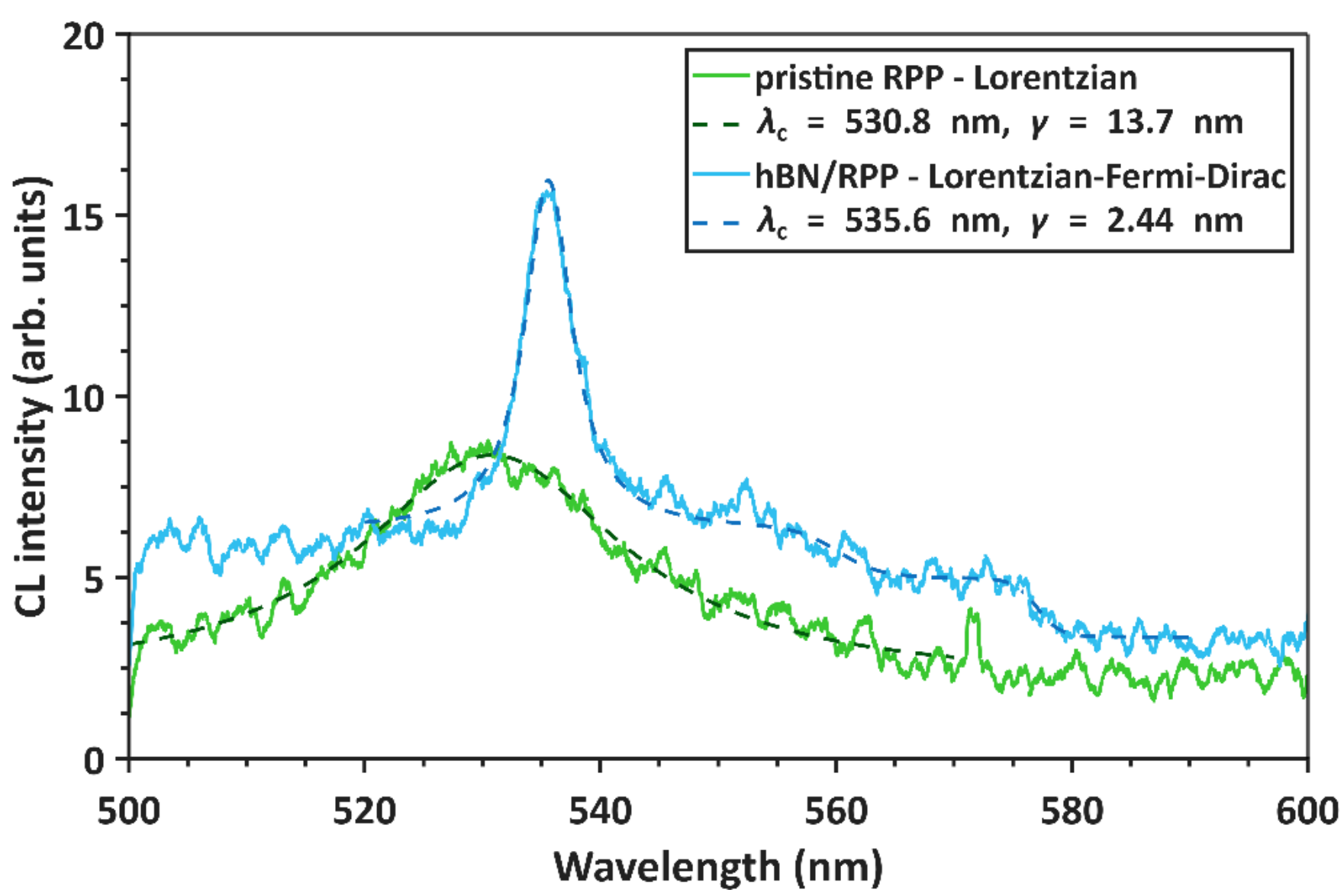

Supplementary Figure 19: **Fiber-collected CL spectra at different excitation spots of the hBN/RPP heterostructure.** Re-plot of the measured CL spectra shown in Figure 1d in the main text, for electron beam excitation positions at the pristine RPP crystal (green) and the stacked hBN/RPP heterostructure, marked by color-coded circles in Figure 1b in the main text. Additionally, Lorentzian functions were fitted to each spectrum (dashed lines) to reveal the central peak positions and their widths. For the heterostructure, the fit also contains Fermi-Dirac functions to include the phonon sidebands of the hBN excitation.

Supplementary Note 9: Investigation of a complementary WS_2/hBN/C heterostructure via mirror-collected cathodoluminescence

Below we show the long-range energy transfer of excitons by coupling to hBN defects, as also observed in WS_2/hBN heterostructures. In the SEM image (Supplementary Figure 20a), a WS_2 flake is shown which is partially covered by an hBN flake. The CL spectra along a line from the blue spot to the purple spot is plotted in panel b – this path is also marked by a black arrow. Along the distance axes, changing from $0\ \mu m$ to $3.25\ \mu m$, the CL is taken from pure WS_2. The CL emission has a peak around $600\ nm$, which is better demonstrated in Supplementary Figure 20c, consistent with exciton A in WS_2 [11]. From $3.25\ \mu m$ to $4.42\ \mu m$, the CL spectra of WS_2/hBN heterostructures show that WS_2 excitons are more dominant than hBN radiative defect centers of $530\ nm$ and $575\ nm$ as observed in pristine hBN shown in Supplementary Figure 20d. The CL intensity increases substantially in the heterostructure region. From $4.42\ \mu m$ onwards, we are moving away from the heterostructure into the extruded part of hBN which causes a shift in CL peaks from WS_2 excitons to hBN defects.

The red spectrum in Supplementary Figure 20c represents the heterostructure region that shows the WS_2 dominated response. The orange spectrum is taken from the beginning of the hBN region with the highest intensity – therefore a combination of CL signatures of both WS_2 and hBN are observable. In the purple spectrum the intensity is reduced and the peaks of pristine hBN become more dominant. The CL spectra of pristine WS_2 and hBN is plotted in Supplementary Figure 20d, whereby the CL signal from WS_2 is substantially weaker compared to the CL emission from hBN. This is in contrast with the measurements with RPP/hBN heterostructure, where the peaks corresponding to RPP excitons are dominant and significantly enhanced compared to the emission from WS_2 excitons. This can be explained by the fact that exciton signal of RPPs were much stronger compared with bulk WS_2, since the oscillator strength of exciton excitation in WS_2 is 0.017 [12], but the oscillator strength of RPP excitons is 0.7 [13]. However, the incoherent coupling of an emitters' excitons and hBN defects is also observed here particularly consistent with what was observed with RPP($n = 2$)/hBN heterostructures (Supplementary Figure 5).

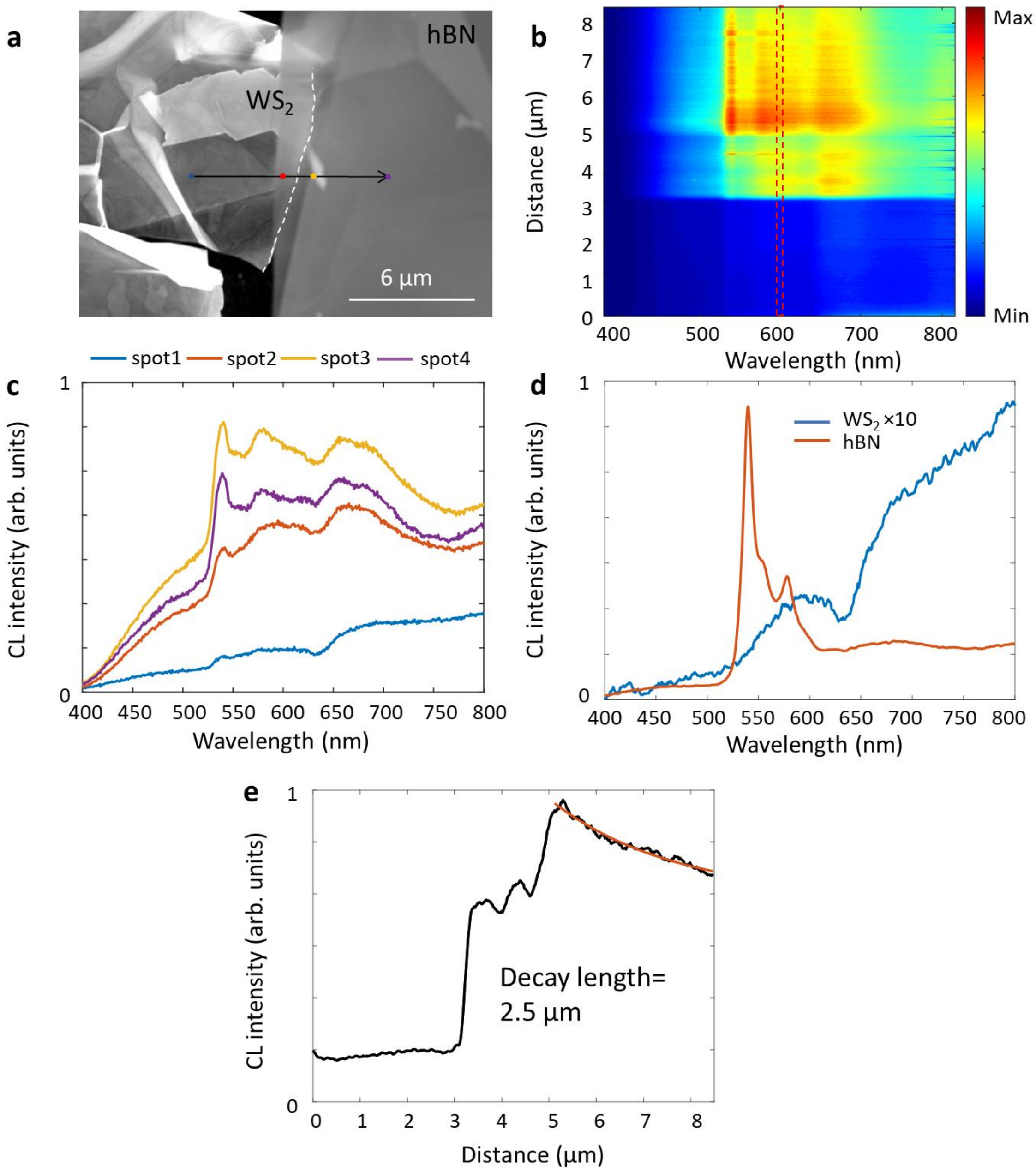

Supplementary Figure 20. (a) SEM image of WS_2 flakes covered by a large hBN sheet. The colored circles show the positions at which the CL spectra in (c) are presented. (b) CL spectra acquired along the arrow shown in panel (a), capturing the substantial enhancement of the CL intensity in the covered regions where hBN and WS_2 overlap, as well as (e) the decay of the CL intensity in the extruded hBN flakes at the length of approximately 2.5 µm. (d) CL spectra of pure WS_2 and hBN flakes, demonstrating the partial emission overlap.

Supplementary Note 10: Complementary photoluminescence measurements of RPP/C and RPP/hBN/C heterostructures

In addition to cathodoluminescence (CL), we performed photoluminescence (PL) measurements using a high-numerical-aperture objective ($NA = 0.9$) to compare optical and electron-beam excitation schemes and to further assess the suitability of CL for probing exciton-defect coupling.

In contrast to CL, which provides highly localized and non-resonant excitation, PL excitation coherently and resonantly addresses a comparatively large ensemble of emitters within the diffraction-limited excitation spot. As a result, PL injects excitation collectively into a substantial portion of the defect network, leading to different redistribution and relaxation dynamics than those initiated by the spatially confined excitation in CL.

A supercontinuum laser source was used for PL excitation at photon energies above the RPP exciton resonance (425 nm-445 nm). PL imaging shows that, for bare RPP flakes (Supplementary Figure 21a), the emission remains largely localized around the excitation spot. In contrast, for hBN-covered RPP heterostructures, the emission becomes spatially more extended across the hBN flake (Supplementary Figure 21), consistent with enhanced coupling between RPP excitons and hBN defect states. Spectrally, we observe enhanced emission intensity from hBN-covered RPP (Supplementary Figure 21d) compared to bare RPP on carbon (Supplementary Figure 21c), in qualitative agreement with the trends observed in CL spectroscopy.

However, the spatial distribution of PL emission differs from the CL results. Whereas CL measurements reveal a relatively uniform and homogeneous intensity decay consistent with an approximately exponential attenuation across the hBN flake, PL images exhibit a more inhomogeneous intensity distribution. This behavior is attributed to coherent optical excitation and multiple light scattering effects from wrinkles and other sample inhomogeneity, which make the PL signal more sensitive to local thickness variations, surface roughness, and other structural inhomogeneities that influence light propagation.

These complementary PL measurements therefore support the presence of coupling between RPP excitons and hBN defects, while also highlighting that CL provides a more localized and controlled excitation geometry for probing energy redistribution within the defect network.

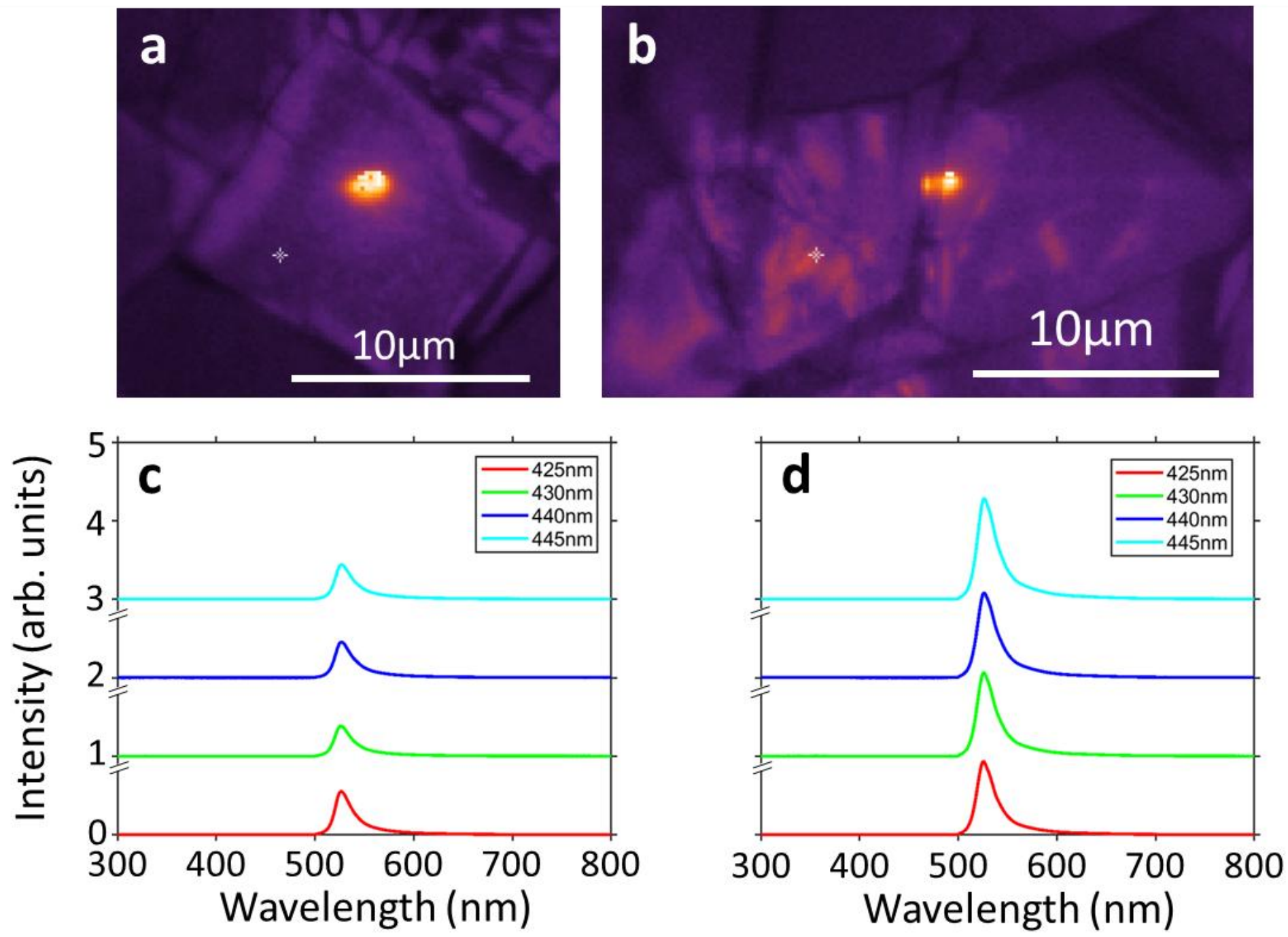

Supplementary Figure 21. **PL spectroscopy and imaging of RPP/C and RPP/hBN/C heterostructures.** The PL images of (a) an RPP/C flake and (b) an hBN/RPP/C heterostructure, where the hot spot at the middle shows the laser excitation location. The excitation wavelength is 480 nm. PL spectra at the excitation location captured from (c) RPP/C and (d) hBN/RPP/C heterostructures.

References

1. Demers, H.; Poirier-Demers, N.; Couture, A., R.; Joly, D.; Guilmain, M.; Jonge, N.; Drouin, D. Three-dimensional Electron Microscopy Simulation with the CASINO Monte Carlo Foftware. *Scanning* **2011**, *33*, 135–146.

2. Demers, H., Poirier-Demers, N.; Phillips, M. R.; Jonge, N.; Drouin, D. Three-Dimensional Electron Energy Deposition Modeling of Cathodoluminescence Emission Near Threading Dislocations in GaN and Electron-Beam Lithography Exposure Parameters for a PMMA Resist. *Microscopy and Microanalysis* **2012**, *18*, 1220–1228.

3. Drouin, D.; Couture, A. R.; Joly, D.; Tastet, X.; Aimez, V.; Gauvin, R. CASINO V2.42—A Fast and Easy-to-use Modeling Tool for Scanning Electron Microscopy and Microanalysis Users. *Scanning* **2007**, *29*, 92–101.

4. Chahshouri, F.; Taleb, M.; Diekmann, F. K.; Rossnagel, K.; Talebi, K. Interaction of Excitons with Cherenkov Radiation in WSe2 Beyond the Non-recoil Approximation." *Journal of Physics D: Applied Physics* **2022,** *55*, 145101.

5. Harrington, R. F. Time-Harmonic Electromagnetic Fields. McGRAW-HILL BOOK COMPANY, New York 1961.

6. Bittorf, P. H.; Black, M.; Crispin, H.; Singh, P.; Darman, P.; Darbari, S.; Chahshouri, F.; Taleb, M.; Talebi, N. Long-Range Exciton Energy Transfer in Two-Dimensional Materials. *Laser Photon. Rev.* **2026**, *20*, e01604.

7. Arakawa, E.; Williams, T. M. W.; Inagaki, T. Optical Properties of Arc-evaporated Carbon Films Between 0.6 and 3.8 eV. *J. Appl. Phys.* **1977**, *48*, 3176-3177.

8. Lee, S. Y.; Jeong, T.-Y.; Jung, S.; Yee, K.-J. Refractive Index Dispersion of Hexagonal Boron Nitride in the Visible and Near-infrared. *Phys. Status Solidi B*, **2018**, *256*, 1800417.

9. Song, B.; Hou, J.; Wang, H.; Sidhik, S.; Miao, J.; Gu, H.; Zhang, H.; Liu, S.; Fakhraai, Z.; Even, J.; Blancon, J.-C.; Mohite, A. D.; Jariwala, D. Determination of Dielectric Functions and Exciton Oscillator Strength of Two-dimensional Hybrid Perovskites. *ACS Mater. Lett.* **2020**, *3*, 148-159.

10. Taleb, M.; Davoodi, F.; Diekmann, F. K.; Rossnagel, K.; Talebi, N. Charting the Exciton–Polariton Landscape of WSe2 Thin Flakes by Cathodoluminescence Spectroscopy. *Adv. Photonics Res.* **2022**, *3*, 2100124.

11. Jung, G.-H.; Park, Q. H. Measuring the Optical Permittivity of Two-dimensional Materials without a *priori* Knowledge of Electronic Transitions. *Nanophotonics* **2019**, *8*, 263-270.

12. Wang, Q.; Sun, L.; Zhang, B.; Chen, C.; Shen, W.; Lu, W. Direct Observation of Strong Light-Exciton Coupling in Thin WS_2 Flakes. *Opt. Express* **2016**, *24*, 7151-7157.

13. Ishihara, T.; Takahashi, J.; Goto, T. Exciton state in two-dimensional perovskite semiconductor $(C_{10}H_{21}NH_3)_2PbI_4$. *Solid State Commun.* **1989**, *69*, 933-936.
